# Quaternary aminostratigraphies for the eastern North European Plain.

**DOI:** 10.12688/openreseurope.21815.1

**Published:** 2025-12-22

**Authors:** Ellie Nelson, Dustin White, Lucy Wheeler, Stefan Meng, Marcin Szymanek, Jaqueline Strahl, Michael Hein, Witold P. Alexandrowicz, Brigitte Urban, Samantha Greeves, Mareike Stahlschmidt, Ralf-Dietrich Kahlke, Tobias Lauer, David Colin Tanner, Kirsty E.H Penkman

**Affiliations:** 1 Department of Chemistry, University of York Department of Chemistry, York, England, YO10 5DD, UK; 2 Institute of Geography and Geology, Universitat Greifswald, Greifswald, Mecklenburg-Vorpommern, Domstraße 11 17489, Germany; 3 Faculty of Geology, University of Warsaw, Warsaw, Masovian Voivodeship, 02-089, Poland; 4 Land Brandenburg Landesamt fur Bergbau Geologie und Rohstoffe, Cottbus, Brandenburg, 03046, Germany; 5 Institute of Ecology, Leuphana University of Lüneburg, Lüneburg, Lower Saxony, 21335, Germany; 6 LeipzigLab - Historical Anthropospheres Working Group, Universitat Leipzig, Leipzig, Saxony, 04107, Germany; 7 AGH University of Science and Technology Faculty of Geology Geophysics and Environmental Protection, Kraków, Lesser Poland Voivodeship, 30-059, Poland; 8 Department of Evolutionary Anthropology, University of Vienna, Wien, 1030, Austria; 9 Human Evolution and Archaeological Sciences (HEAS), University of Vienna, Wien, 1030, Austria; 10 Senckenberg Research Station of Quaternary Period Palaeontology, Weimar, Thuringia, D-99423, Germany; 11 Terrestrial Sedimentology, Department of Geosciences, Eberhard Karls Universitat Tubingen, Tübingen, Baden-Württemberg, 72076, Germany; 12 LIAG Institute for Applied Physics, Hannover, 30655, Germany

**Keywords:** aminostratigraphy, geochronology, pollen stratigraphy, Middle Pleistocene

## Abstract

The eastern North European Plain is an important area for studying Quaternary climate change and archaeology; however, providing chronological constraints for deposits can be challenging. Amino acid geochronology (AAG) is a relative dating technique that has been useful in correlating isolated Quaternary deposits. The intra-crystalline protein decomposition (IcPD) approach to AAG using the opercula of *Bithynia* snails has previously been used to provide relative dating frameworks across northern and central Europe in areas where the integrated diagenetic temperature can be assumed to be similar. Here, the first aminostratigraphies for the eastern North European Plain are presented, incorporating deposits from at least the last ~1 Ma, which are used to assess the current age attributions to Middle and Late Pleistocene interglacials. These aminostratigraphies are then used to explore expected differences in the extent of IcPD due to differing temperature histories across the study area. Correlations of opercula to regional pollen assemblages representative of the Holsteinian, Eemian and Holocene are used to evaluate the temporal resolution achievable by IcPD within a given interglacial. This work has produced four new aminostratigraphies that can now be used as reference datasets for relative age estimation for the late Middle Pleistocene to the Holocene in the eastern North European Plain.

## Plain language summary

The eastern North European Plain is an important region for studying past climate change over the last million years. In this area, the climate has cycled between cold periods (when ice sheets covered most of the region) and warm periods (when sea levels were much higher than today). Records of these changes are preserved in sediments, but to understand when and how these dramatic shifts in climate occurred, these need to be dated. This can be challenging, as most dating techniques do not extend this far back in time, or the sediments lack the material required for dating. In this study, we have used amino acid geochronology on fossil snails to place these sediments in relative order of age and to narrow down the period during which they were deposited. The work establishes four new chronological frameworks that can be used as references for future studies of the Quaternary period (the last 2.6 million years). This will enable us to better understand the effects of past climate change in the eastern North European Plain on the animals and plants that existed during this time, including Palaeolithic humans.

## 1 Introduction

**Figure 1.**
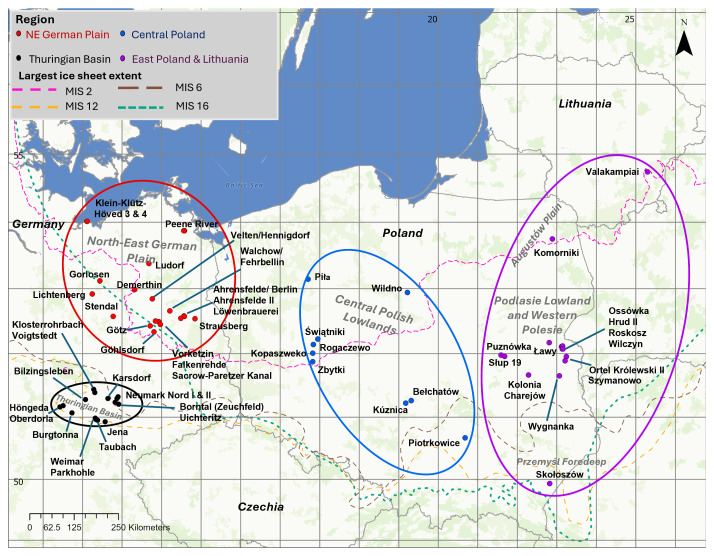
Sites from which opercula analysed in this paper were collected. Sites are grouped into four regions: NE German Plain (red), the Thuringian Basin (black), central Poland (blue), and east Poland & Lithuania (purple). Hypothesised reconstructions, as synthesised by Batchelor *et al.* (2019), of the greatest ice sheet extents of the four largest northern hemisphere glaciations are shown in green (Don/MIS 16), yellow (Elsterian/MIS 12), orange (Saalian/MIS 6), and pink (Weichselian/MIS 2).

The eastern North European Plain is an area of low elevation that is bounded by the Baltic Sea to the north and central European low mountain ranges to the south, covering the northern areas of Germany and Poland; an area from 10°E to 26°E, and 50.5°N to 55°N ( Figure 1). Since the Mid-Pleistocene Transition (~1.2-0.8 Ma; e.g. Legrain *et al.*, 2023), this area has been subjected to multiple glaciations (e.g. Batchelor *et al.*, 2019; Böse *et al.*, 2012; Ehlers *et al.*, 2011; Marks *et al.*, 2019), and was covered in large ice sheets during glacial periods from marine oxygen isotope stage (MIS) 16 to MIS 2 ( Figure 1; Batchelor *et al.*, 2019). The region is characterised by a high potential to archive several decametres of Pleistocene deposits across vast expanses and by the availability of numerous open-cast mines (especially for lignites and for the aggregates industry) exposing these deposits. For these reasons, it is an important region for the study of Quaternary climate change, as well as archaeological sites that can shed light on human adaptations to the changing conditions of the Middle and Late Pleistocene (e.g. Behm-Blancke, 1960; Cyrek *et al.*, 2014; Veil *et al.*, 1994; Vlček *et al.*, 1993). Constraining the chronology of this region is challenging due to the erosional processes that occurred as a consequence of ice sheet expansion and thawing, resulting in Quaternary deposits that can be spatially isolated, redeposited and often only represent a relatively brief snapshot in time (reviewed in Marks, 2023). Therefore, chronostratigraphic methods are required that can cross-correlate these deposits (particularly when fragmentary or only unassignable, insignificant pollen spectra are present) to build a more complete picture of the Quaternary.

Although much progress has been made to understand how regional interglacial deposits correlate with one another (e.g. Cohen & Gibbard, 2022; Marks *et al.*, 2024), the chronostratigraphy of Europe is highly complex and the correlation of regional deposits to global climate is often unclear. The timing of the Eemian interglacial is relatively well-constrained (~130–116 ka; MIS 5e; e.g. Cohen & Gibbard, 2022; Govin *et al.*, 2015; Past Interglacials Working Group of PAGES, 2016; Shackleton *et al.*, 2003; Tzedakis, 2003; Williams *et al.*, 2015), and the Holocene interglacial (~11.7 ka to present, MIS 1; e.g. Walker *et al.*, 2008) is universally agreed ( Cohen & Gibbard, 2022), but the timing of pre-Eemian interglacials is still highly debated (e.g. Gegg *et al.*, 2024; Geyh & Müller, 2005; Nitychoruk *et al.*, 2006; Roe *et al.*, 2009; Scourse *et al.*, 1999). The Holsteinian Interglacial, for example, is generally attributed to MIS 11 (e.g. Cohen & Gibbard, 2022; Lauer & Weiss, 2018). However, the Subcommission for Quaternary Stratigraphy of Germany still officially lists the Holsteinian in MIS 9 (e.g. Stephan, 2014), using the ^230^Th/U ages published by Geyh and Müller (2005). Interglacial stages within the European record are defined by a characteristic tree pollen succession (e.g. Erd, 1973; Krupiński, 1995; Menke & Tynni, 1984; Turner, 1970; West, 1956; Zagwijn, 1996) and pollen successions can be correlated to one another to aid in the identification of an interglacial. However, sedimentary sections containing fragmentary pollen successions, pollen from pre- or post-temperate zones, or interstadial pollen deposits, can make correlation with warm stages within the Quaternary period challenging (e.g. Bridgland, 1994; Roe *et al.*, 2009). It is therefore important to combine other stratigraphic and chronological approaches with pollen stratigraphy in order to correlate Quaternary deposits across regions.

Amino acid geochronology (AAG) is a relative dating technique which has been useful in correlating isolated Quaternary deposits within a region where a similar temperature history can be assumed (e.g. Hearty & Aharon, 1988; Miller & Mangerud, 1985; Ortiz *et al.*, 2004; Penkman *et al.*, 2011; Penkman *et al.*, 2013; Tesakov *et al.*, 2020; Wehmiller, 1982). The intra-crystalline protein decomposition (IcPD) approach to AAG using the opercula of *Bithynia* snails has been demonstrated to be effective in cross-correlating Quaternary sites in Britain ( Penkman *et al.*, 2011; Penkman *et al.*, 2013; Preece *et al.*, 2020), the Eastern European Plain ( Tesakov *et al.*, 2020), and the northern Upper Rhine Graben, Germany ( Nelson *et al.*, 2025a). Where both opercula and pollen are present in the same stratigraphic horizons, IcPD analysis can be used alongside pollen stratigraphy to confirm age correlations to chronostratigraphic stages within the Quaternary (e.g. Gegg *et al.*, 2024; Nelson *et al.*, 2025a; Penkman *et al.*, 2013), but this approach has yet to be applied to northern continental Europe. In addition, the timing within an interglacial indicated by the pollen present can be used to assess the resolution achievable for a given region by IcPD.

In this study, four new regional aminostratigraphies from the east North European Plain are presented: the north-east (NE) German Plain, Thuringian Basin, central Poland and east Poland & Lithuania ( Figure 1). They are used to assess the current age attributions of these late Early, Middle and Late Pleistocene sites, and explore any systematic differences in IcPD due to differing temperature histories between the regions (e.g. Miller & Mangerud, 1985; Wehmiller, 1982). The temporal resolution that can be achieved by IcPD within an interglacial is also examined using correlated regional pollen zones for Holsteinian ( Erd, 1973; Müller, 1974a; Müller, 1974b; Strahl, 2023)/Mazovian ( Bińka & Nitychoruk, 1995; Bińka *et al.*, 1997; Krupiński, 1995), Eemian ( Behre & Lade, 1986; Cheddadi *et al.*, 1998; Erd, 1973; Hermsdorf & Strahl, 2008; Menke & Tynni, 1984; Turner, 2002; Zagwijn, 1996) and Holocene ( Mangerud *et al.*, 1974; Sernander, 1908, reviewed in Birks and Seppä, 2010) aged material. This work provides new reference aminostratigraphies that can be used to constrain the chronology of Quaternary deposits within the eastern North European Plain, providing an additional tool to date this region’s climatic and environmental past.

## 2 The geological, chronological and climate context of the eastern North European Plain

**Figure 2.**
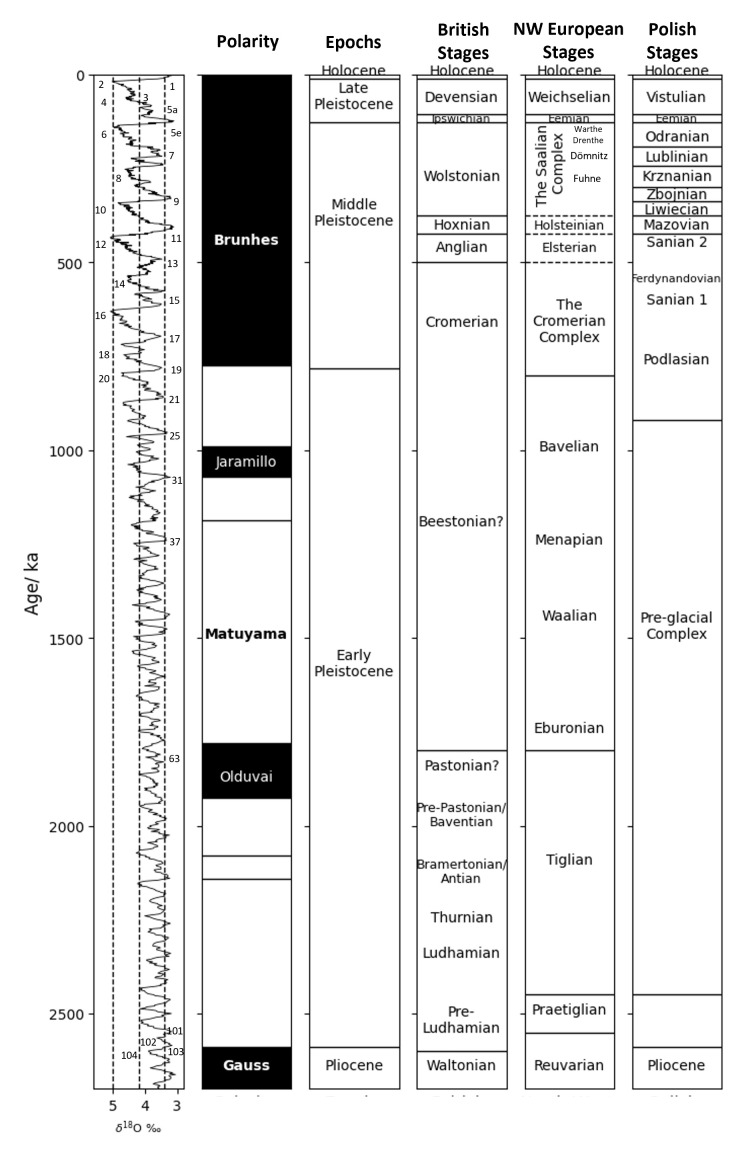
Chrono-stratigraphical subdivision of the Quaternary (from left to right): marine oxygen isotope stages (MIS), palaeomagnetic record (black= normal polarity, white = reversed polarity), epochs, standard stages, British stages, North-West European stages (Germany), and Polish stages. Figure has been recreated from the summary of northern European Quaternary chronostratigraphy correlations by Szymanek and Julien (2018) and Cohen and Gibbard, 2022. German sub-stages of the Saalian Complex have been correlated according to Bittman *et al.* (2018; Table 1). Note disagreement between the different alignments for the Cromerian Complex, Elsterian, Holsteinian and Saalian Complex in the NW European chronostratigraphy, represented by dashed lines.

Sites for all regions covered in this study were selected for the presence of bithyniid opercula associated with independent chronostratigraphic evidence. This includes palynology, molluscan and mammalian biostratigraphy, fluvial terrace sequences, palaeomagnetic boundaries, ESR and U/Th series and luminescence dating (see SI). Nearly all the sites included in this study are correlated with chronostratigraphic stages from the last 1 million years ( Figure 2; SI), and most have independent evidence of age. A summary of regional terminologies and the generally agreed correlations between regional chronostratigraphies and the MIS record is presented in Figure 2. It should be noted that these correlations may be the result of different research traditions across Europe, and although progress has been made to cross-correlate regional chronostratigraphic stages with each other and the MIS record, age attributions of various sites/regional Quaternary warm stages may differ. The North-Western European terminology as outlined by Cohen and Gibbard (2022) will be used throughout, but regional terminology will also be used where relevant.

Opercula were recovered from the surface to depths of 75 m below ground level, as this is above the depth at which systematic IcPD differences due to geothermal warming were observed in the Pannonian Basin ( Nelson *et al.*, 2024). The Pannonian Basin has a higher surface heat flow compared to the regions included in this study ( Cloetingh *et al.*, 2010), so in material presently buried <75 m below the surface we assume that climate has been the most significant influence on the integrated temperature experienced by the opercula used in this study (e.g. Nelson *et al.*, 2024; Nelson *et al.*, 2025a; Wehmiller & Stecher, 2000).

### 2.1 Variability in integrated burial temperatures across the east North European Plain

**Figure 3.**
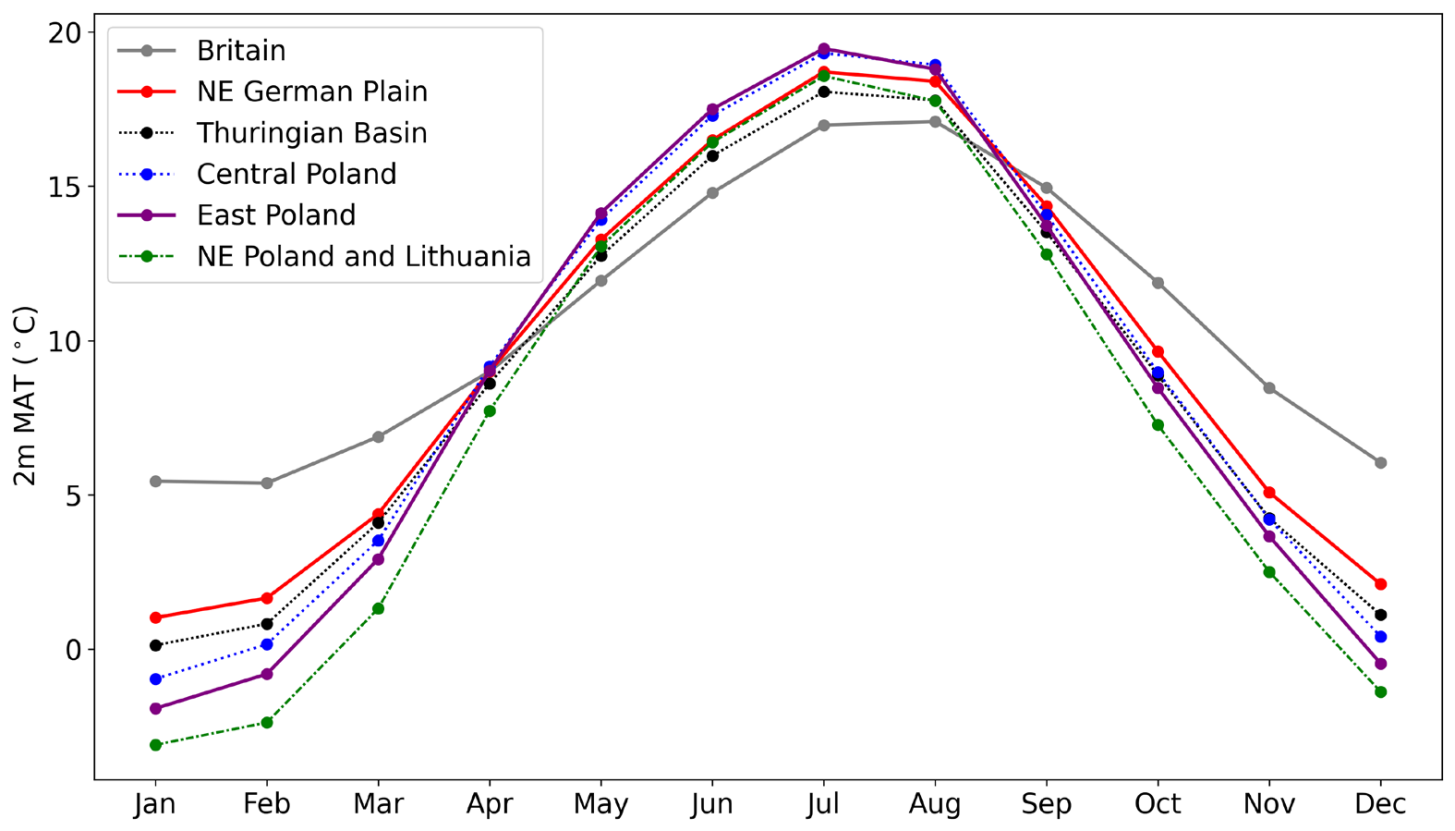
The monthly mean temperature between 1991–2020 (ERA5; Hersbach *et al.*, 2020) for all regions included in this study. British average temperatures are for the southern half of England, the location of most British IcPD samples ( Penkman *et al.*, 2011; Penkman *et al.*, 2013). Note the smaller variation between seasonal temperatures in more maritime climates (e.g. Britain) compared to more continental climates (e.g. Poland).

The intra-crystalline region of an operculum acts as a closed system; therefore, the assumption is that the only variables that can affect extent of IcPD is temperature and time since biomineralization. In this closed system, there is no evidence that local environmental conditions - such as sediment types, water content, pH etc. - have an impact on the extent of IcPD in non-remineralised opercula (e.g. Nelson *et al.*, 2024; Nelson *et al.*, 2025a; Penkman *et al.*, 2011; Penkman *et al.*, 2013; Penkman *et al.*, 2024; Preece *et al.*, 2020; Tesakov *et al.*, 2020). Therefore, for the extent of IcPD analysis to be directly comparable between sites, a similar temperature must have been experienced throughout the burial history. For fossils buried near the surface, burial temperature is largely influenced by the climate (e.g. Nelson *et al.*, 2024; Nelson *et al.*, 2025a; Wehmiller, 1982). At present, the east North European Plain is associated with a humid continental climate ( Beck *et al.*, 2018). Present mean annual surface temperature (MAT) in the study area ranges from 7.5 to 9.5°C; the temperature difference between the coldest and warmest months ranges between ~18–22°C (ERA5; Hersbach *et al.*, 2020; Table 1; Figure 3). Continentality increases from west to east and north to south, as distance from the Atlantic Ocean and Baltic Sea increases (e.g. Beck *et al.*, 2018). Therefore, integrated burial temperatures will likely vary throughout this region and will have done so throughout the Quaternary.

**Table 1.** Mean annual temperature (2 m above surface) and the difference between the warmest and coldest month for the regions included in this study from 1991 to 2020 (ERA5; Hersbach *et al.*, 2020). British average temperatures are for the southern half of England, the location of most British IcPD samples in the reference dataset ( Penkman *et al.*, 2011; Penkman *et al.*, 2013).

Region	Mean annual temperature (°C)	Mean temperature of the warmest month (°C)	Mean temperature of the coldest month (°C)	Difference between warmest and coldest month (°C)
Britain	10.7	17.0	5.4	11.6
Thuringian Basin	8.8	18.1	0.1	18.0
NE German Plain	9.5	18.7	1.0	17.7
Central Poland	9.1	19.3	-1.0	20.3
East Poland	8.7	19.5	-1.9	21.4
NE Poland & Lithuania	7.5	18.6	-3.1	21.7

To confirm this, sites were grouped into four sub-regions, which were defined by their geographic area (described in the next section) and similarity in MAT and annual temperature range. The extent of IcPD between chronostratigraphic stages is compared between each sub-region. In addition, IcPD results will be compared to the nearby British aminostratigraphy, where the range of ᴅ/ʟ values ( Section 3.2.2) for warm marine oxygen isotope stages (MIS) is well established by independent chronology ( Penkman *et al.*, 2011; Penkman *et al.*, 2013). Britain has a more oceanic climate with warmer, wetter winters and cooler summers ( Met Office, 2016). The British Isles have a higher MAT compared to the NE continental European sites, but smaller seasonal differences in temperature ( Figure 3; Table 1). Understanding the variability in burial temperature will be key to start to correct for this and enable cross-correlation of sites across Europe.

### 2.2 Geological context

In this study, aminostratigraphic frameworks from four regions in north-east continental Europe were developed: the NE German Plain and Thuringian Basin in Germany, central Poland, and east Poland & Lithuania ( Figure 1; Table 1). All regions occur within or in relatively close proximity to the North European Plain, which is an area of low-lying land between the North and Baltic Seas and Central European Uplands (reviewed in Woronko & Dąbski, 2022). It is a transitional area between oceanic and continental climates and has been exposed to repeated glaciations in the last 500 ka. All sites also lie within the margins of the largest ice sheet extents of the Elsterian and/or Saalian glacial episodes ( Batchelor *et al.*, 2019). As such, the chronology of these regions is critical for understanding Quaternary climate.

***2.2.1 North-East German plain***

The North German Plain covers the entirety of northern Germany and is bounded by the Central German Uplands to the south. Its current topography is largely due to glacial processes of the Middle to Late Pleistocene (e.g. Stackebrandt, 2009), with the area crossed by the Elsterian, Saalian and Weichselian glaciers (e.g. Batchelor *et al.*, 2019; Ehlers *et al.*, 2004). The north-east of this region contains a multitude of lakes (the Mecklenburg Lake Plateau) which were formed during the retreat of the Weichselian ice sheet. The south-westerly areas are drier, and more weathered and levelled as a result of erosion from the Saalian ice sheet and posterior periglacial processes. The area is mainly drained by the Elbe and Oder rivers, which flow to the North and Baltic Seas.

***2.2.2 Thuringian Basin and surrounding areas***

The Thuringian Basin lies within central Germany. It is surrounded by areas of high elevation, including the Harz Mountains to the north, the Thüringer Wald to the southwest, the Thuringian Slate Mountains to the southeast and Eichsfeld to the west. The underlying bedrock of the basin is predominantly formed of Triassic sedimentary rocks including Muschelkalk and Keuper marl; in contrast the surrounding mountains are largely formed of Palaeozoic rocks (reviewed in Kramm *et al.*, 2020). The basin itself lies 200–300 m above sea level and within the Central European climate region, but due to its confinement within areas of high relief it exhibits strong micro- and meso-climatic variability and below-average precipitation values (summarised in Meyrick & Schreve, 2002). Only the glaciers of the Elsterian glaciation reached the northeastern edge of the area. This region has a rich history of Quaternary research (reviewed in Kahlke, 2002) and is particularly renowned for its Quaternary mammalian and molluscan fossils (e.g. Bratlund, 1999; Kahlke, 1965; Kahlke, 1969; Kahlke, 1974; Kahlke, 1975; Kahlke, 1977; Kahlke, 1978; Kahlke, 1984; Mania, 1973; van Kolfschoten, 2000; Wiefel, 1977). The type sites for the pre-Elsterian Borntal (warm stage between Tiglian and Waalian; Mania, 1973) and Artern (MIS 29-21; Maul *et al.*, 2013) interglacials are all found here.

***2.2.3 Central Poland***

All sites in this region are situated on the Central Polish Lowlands, also known as the “Polish Plain”, which is a low-lying area in the central-western part of Poland. They are bordered to the north by the coastal plain, and to the south by the Polish Highlands. This region is situated within the maximum ice extent of the Sanian 1 & 2, Odranian and Vistulian glaciations (see Figure 2 for correlations with MIS record and NW European stages; see Figure 1 for greatest ice sheet extents). As such, much of the terrain has been shaped by glacial processes, resulting in pro-glacial lakes which are now filled with sediment, and low hills in otherwise flat terrain (e.g. Marks *et al.*, 2016).

***2.2.4 East Poland & Lithuania***

Three separate regions have been categorised as east Poland & Lithuania due to the sparsity of sites in some localities. The majority of sites are located in the Podlasie Lowland and Western Polesie. This is an area located in the east of Poland on the border with Belarus and Ukraine, to the north of the Lublin Upland. The remnants of a Holsteinian/ Mazovian (e.g. Albrycht *et al.*, 1997; Nitychoruk, 1994; Nitychoruk, 2000) and Eemian Lakeland ( Hrynowiecka *et al.*, 2018) are preserved in this region. Both sets of lake deposits are a result of ice-retreat of the preceding glacial episode, with lakes forming in depressions within surface tills. The region now features varied relief, with numerous lacustrine plains and peat bogs (e.g. Marks *et al.*, 2016; Pochocka-Szwarc *et al.*, 2024).

Three sites fall outside this: Komorniki is located in the Augustów Plain, in the north-east of Poland; it is a vast glacial plain that has been covered by ice sheets at multiple points over the last 1 Ma (e.g. Batchelor *et al.*, 2019; Ber *et al.*, 2006). Valakampiai is in Lithuania and is considered the type section for the Snaigupélè Interglacial ( Kondratienė, 1959), which is commonly attributed to MIS 7 ( Molodkov *et al.*, 2002). The more southerly site of Skołoszów is located in the Przemyśl Foredeep; an area located between the Lesser Poland Upland, Lublin Upland and Wester Carpathian. These three sites have been added to the “East Poland & Lithuania” aminostratigraphic framework as the Podlasie Lowlands and Western Polesie have the most similar modern MAT and seasonal temperatures to these sites (see Section 2.2). All sites in this regional framework are situated ~100 m above sea level. It should be noted that these three regions may not be directly compared due to regional variation in climate, and therefore MAT, between the north and south. Lithuania and Augustów Plain are currently on average 1°C colder ( Table 1; Figure 3) than the Podlasie Lowland, Western Polesie and Przemyśl Foredeep. Should a similar temperature difference have occurred throughout the Quaternary this may impact the extent of IcPD.

## 3 Materials and methods

### 3.1 Materials

To build the regional frameworks using IcPD, analysis of bithyniid opercula ranging in age from the Bavelian (~1.1-0.78 Ma; Cohen & Gibbard, 2022; Zagwijn & de Jong, 1984) to the Holocene (~11.7 ka) was carried out across the 4 regions. Opercula from one site attributed to the Miocene (~20–5 Ma) was also analysed to confirm whether endogenous intra-crystalline amino acids were still preserved in opercula of this age. It should be noted that bithyniids are a Palaearctic species and are not known from full glacial deposits (e.g. Alexandrowicz, 1999; Johansen, 1904; Penkman *et al.*, 2013), therefore in this area IcPD values are likely to only represent warm stages/substages from this last 1 Ma.

Bithyniid opercula were analysed from 60 sites and boreholes from across north-east continental Europe (see SI) to build the aminostratigraphies developed in this study. The majority of specimens have been identified as *Bithynia tentaculata* (Linnaeus, 1758), however, several other species of the genus *Bithynia* analysed as pilot data indicate that they produce statistically similar extents of IcPD for samples of equivalent age ( Penkman *et al.*, 2007; Penkman *et al.*, 2013; Tesakov *et al.*, 2020). This includes *Bithynia troschelii*/ *transsilvanica* (Paasch, 1842), *Bithynia* cf. *bavelensis* ( Meijer, 1990), and specimens which have been identified to genus level only. Opercula from *Parafossarulus crassitesta* ( Brömme, 1885) and the genus *Pomatias* have also been used.

**Table 2. (Available in the SI)** The context of the opercula samples analysed in this study. Individual opercula (between 1 to 9 whole opercula or opercula fragments were selected) were analysed from a stratigraphic level within a sediment profile or borehole from a given location. The region, site or borehole name, sample depth (where recorded) or stratigraphic unit, morphology, other independent evidence of age, and associated pollen zone is described. The ‘NEaar no.’ is the identification number for each individual fossil sample analysed in the NEaar Laboratory, University of York. Please note that the pollen zonation (PZ) systems used to define pollen correlated with opercula in this study have been described in the SI (see Section 7.1: tables S1–S3, and SI 7.3; N/A = no pollen sequences available for correlation). Due to the variability between different pollen classification systems and to allow for cross-comparison between regional pollen stratigraphies, in this paper the Holsteinian and Eemian interglacials have been divided into four sub-periods: the Pre-temperate, Early-temperate, Late-temperate and post-temperate biostratigraphic zones (according to Turner & West, 1968). The Holocene has been divided into the Upper, Middle and Lower substages.

### 3.2 Methods

***3.2.1 Intra-crystalline protein decomposition method***

All samples were prepared using the procedure outlined for determining IcPD by Penkman *et al.* (2008) (for full methods see SI). This involves the isolation of the intra-crystalline fraction by oxidation (48 h, 12% NaOCl), followed by the division of the sample into two fractions: the FAA fraction (FAA, naturally occuring free amino acids as a result of protein degradation) and total hydrolysable amino acid fraction (THAA). Both FAA and THAA fractions were analysed in analytical duplicate using a modified version of the reverse-phased high-performance liquid chromatography (RP-HPLC) method described by Kaufman and Manley (1998), along with standards and blanks.

***3.2.2 Intra-crystalline protein decomposition method***

Three key reaction pathways occur during protein decomposition following the cessation of tissue turnover in an organism. The first two are the hydrolysis of the peptide bond and decomposition to other more stable amino acids and smaller organic molecules ( Bada *et al.*, 1978; Kossiakoff, 1988; Penkman *et al.*, 2008; Sato *et al.*, 2004). In addition, spontaneous racemisation occurs resulting in a slow change from only the ʟ-isomers to a racemic equilibrium of ʟ and ᴅ forms; this ratio is quantified as the ᴅ/ʟ value. Amino acids are chiral molecules that exist in two mirror-image forms, but only the ʟ-isomers (left-handed) are naturally produced and incorporated into living proteins, making the progressive conversion to ᴅ-forms (right-handed) a useful indicator of post-mortem molecular alteration. This is quantified as the ᴅ/ʟ value. In a closed system, the FAA and THAA ᴅ/ʟ values should be highly correlated for each amino acid ( Miller *et al.*, 2000), and lack of correlation helps to identify compromised samples ( Preece & Penkman, 2005). The best chromatographically resolved amino acid pairs using RP-HPLC in opercula are serine (Ser), aspartic acid/asparagine (Asx), alanine (Ala), glutamine/glutamic acid (Glx), and valine (Val) ( Penkman *et al.*, 2013; Powell *et al.*, 2013), so the analysis will focus on these amino acids. It must be noted that during hydrolysis both asparagine and glutamine can undergo deamidation to aspartic acid and glutamic acid respectively. Therefore, it is not possible to differentiate between these amino acids, so they are referred to as Asx and Glx respectively ( Hill, 1965). They differ in terms of detectability and preservation/stability, and they also racemise at different rates (fastest to slowest: Ser>Asx>Ala>Glx Val; Penkman *et al.*, 2013; Tesakov *et al.*, 2020), providing better temporal resolution over different timescales, and complementary information about age consistencies, preservation and diagenetic history (e.g. Goodfriend, 1991). Ser is geochemically unstable compared to other amino acids, decomposing to alanine in addition to other organic molecules ( Bada *et al.*, 1978). As such, it is not a useful geochronometer for the Pleistocene but is useful as a marker for contamination (e.g. Kosnik & Kaufman, 2008), and to provide temporal resolution for younger specimens attributed to the Holocene (e.g. Conti *et al.*, 2024). Therefore, it has not been used here to evaluate the age of the opercula analysed in this study.

To develop the new regional aminostratigraphies, the data was first screened for non-closed system behaviour ( Preece & Penkman, 2005). Samples where closed-system behaviour had been lost were excluded from further data analysis. In addition, samples where the concentration of all amino acids were below the limit of detection by RP-HPLC were also removed. All these outliers (4%) are detailed in the Sup. Mat.

**Figure 4.**
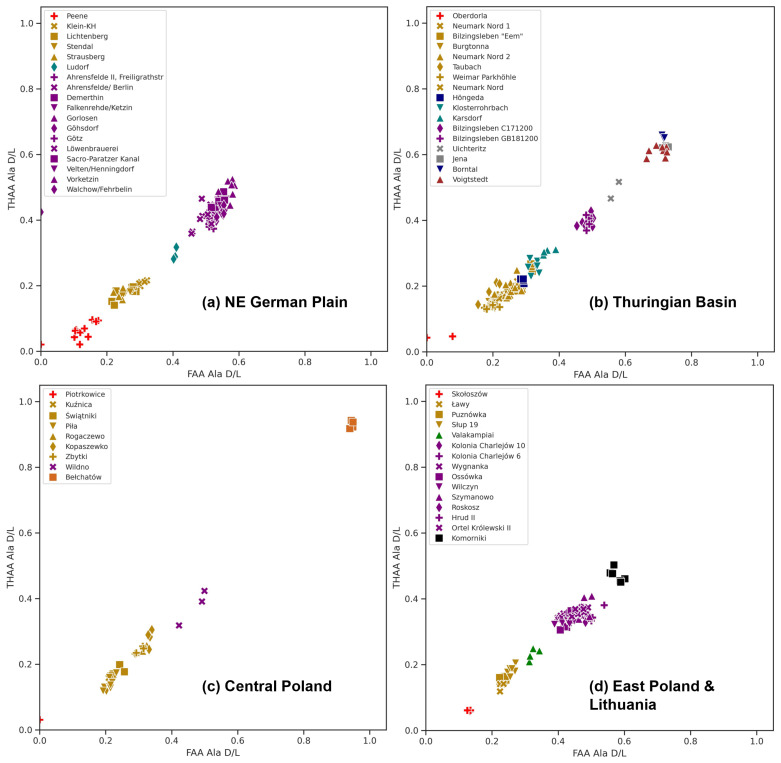
The Ala FAA and THAA fractions from the *Bithynia* opercula analysed for this study after data screening (see SI for samples rejected from this analysis) are presented for each aminostratigraphy: **a**) NE German Plain, **b**) Thuringian Basin, **c**) central Poland, and **d**) east Poland & Lithuania. The youngest material falls to the bottom left of the plot and the oldest to the top right. Horizons are coloured according to their chronostratigraphic attribution as follows: Holocene = red, Eemian = yellow, MIS 7 = green, the Saalian Complex = teal, Middle Pleistocene = blue, Holsteinian = purple, Elsterian = grey, Arternian = dark red, Bavelian = black, and Miocene = dark orange.

Data analysis was performed using Google Colab with Python (3.10). Sample results have been colour-coded according to independent evidence of age ( Figure 4– Figure 8): Holocene = red, the last glacial complex (Vistulian/ Weichselian) = orange, the Last Interglacial (MIS 5e/ Eemian) = yellow, MIS 7 = green, the Saalian Complex = teal, Middle Pleistocene = blue, Holsteinian/ Mazovian = purple, Elsterian = grey, Arternian (MIS 29-21) = dark red, Bavelian = black, Miocene = dark orange. Where the same chronostratigraphic stage is represented in multiple regions, different shades of the same colour are used.

**Figure 5.**
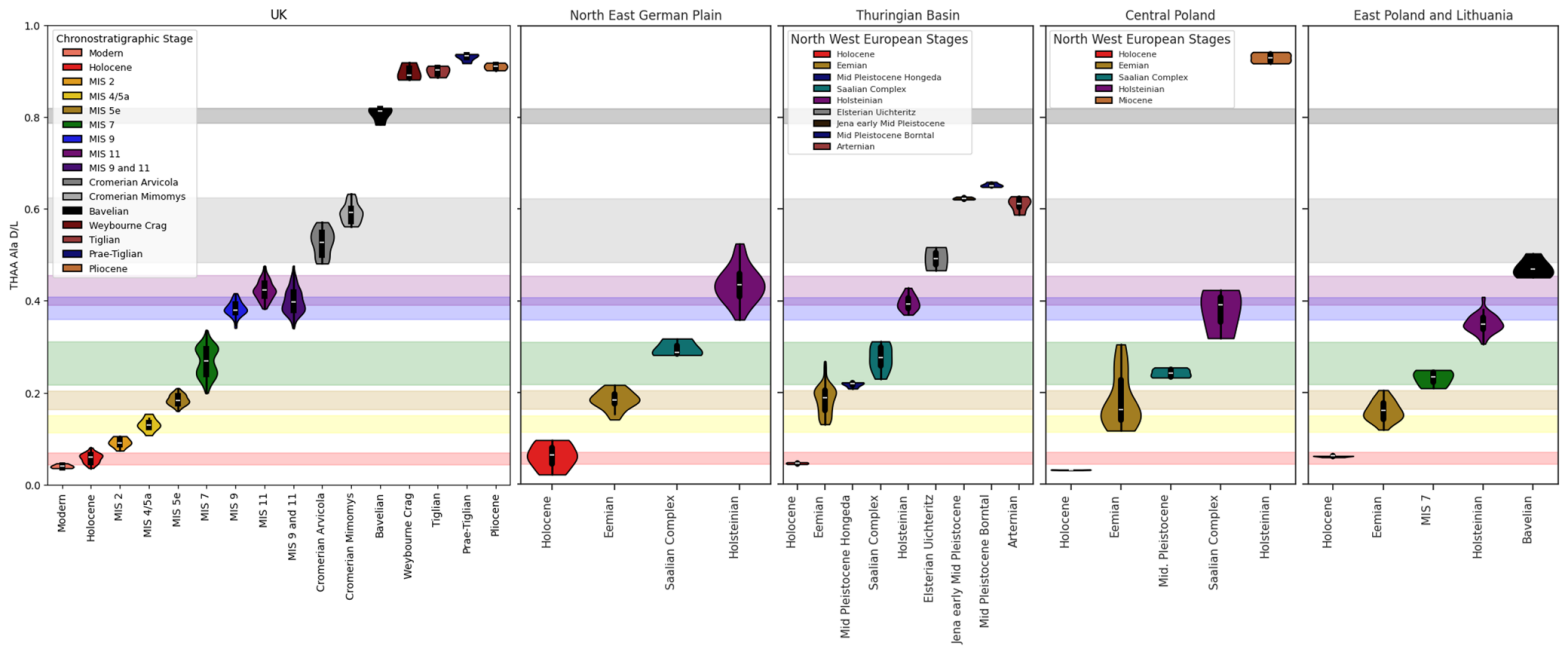
THAA Ala ᴅ/ʟ values for: **a**) Britain ( Penkman *et al.*, 2013: Supplementary Data), **b**) North-East German Plain, **c**) Thuringian Basin, **d**) central Poland, and **e**) east Poland & Lithuania. The 95th percentile range for the British record is demonstrated by horizontal colour bands on each plot, so the ᴅ/ʟ values for all regions can be compared to the British record. In general, for each chronostratigraphic stage, the ᴅ/ʟ values for interglacials assumed to be the same in age decrease from west to east, parallel to increasing continentality. This occurs in all amino acids (see SI).

The range of ᴅ/ʟ values for each chronostratigraphic stage represented in the new aminostratigraphies for the eastern Northern European Plain were compared to the British Quaternary aminostratigraphic framework developed by Penkman *et al.* (2013). A list and map of all sites included is presented in the SI. All information relating to the sites used in this framework, including independent chronology and relevant references, can be found in the Penkman *et al.* (2013): Supplementary Data.

## 4 Results and discussion

### 4.1 IcPD vs. independent evidence of age

After data screening, the ᴅ/ʟ values were compared across all sites for each of the following amino acids: Asx, Glx, Ala and Val ( Figure 4; for Asx, Glx and Val see SI). For each region, ᴅ/ʟ values for sites broadly fell in the expected order given their independent evidence of age. The opercula with the least racemised ᴅ/ʟ values were associated with the Holocene, while specimens that produced the highest ᴅ/ʟ values were from Bełchatów and attributed to the Miocene ( Kowalski & Rzebik-Kowalska, 2002). The Miocene opercula from central Poland were substantially more racemised than the sites with the second highest ᴅ/ʟ values; these came from the Voigtstedt (Hackelsberg), Borntal, Uichteritz, and Jena, all situated in the Thuringian Basin. In the slowest racemising amino acids (Val and Glx) the ᴅ/ʟ values for these Thuringian Basin sites do not exceed 0.5; this implies that IcPD can temporally resolve deposits greater than ~1 Ma from this region.

Except for the Holocene, the mean ᴅ/ʟ values of material from Poland are lower than material from deposits attributed to the same age in Germany ( Figure 4 & Figure 5). This systematic difference in IcPD increases with sample age. For example, the second oldest site, Komorniki (Augustów Plain), attributed to the Bavelian (MIS 31, Ber, 2006; Khursevich *et al.*, 2005), produced lower or similar ᴅ/ʟ values compared to the German Thuringian Basin site attributed to MIS 29-21 (Voigtstedt (Hackelsberg), Arternian Interglacial; Maul *et al.*, 2013).

There are also a few chronostratigraphic stages in each regional record that have a large range of ᴅ/ʟ values when compared to clusters of ᴅ/ʟ values for other stages (see SI). These include Eemian age deposits from central Poland and Holsteinian deposits from the NE German Plain. To understand these patterns, the ᴅ/ʟ values are compared to the ᴅ/ʟ clusters defined for each warm MIS stage within the British aminostratigraphy, where the range of ᴅ/ʟ values for each stage is well established by robust independent chronology ( Penkman *et al.*, 2011; Penkman *et al.*, 2013; Figure 5). In general, the THAA Ala ᴅ/ʟ values from the NE German Plain and Thuringian Basin opercula fall within a similar range to the British MIS stages they are generally assumed to correlate with. However, the mean THAA Ala ᴅ/ʟ values for each Polish Pleistocene chronostratigraphic stage are lower than both the equivalent British and German stages. The differences observed in the ᴅ/ʟ values for each sub-region are discussed in the following sections.

***4.1.1 Eemian and Weichselian***

The Eemian Interglacial was the last interglacial to occur prior to the present day, dated to between ~130–115 ka (e.g. Cohen & Gibbard, 2022). 20 sites in this dataset have been attributed to the Eemian, with many including multiple horizons from the same stratigraphic profile. The mean Ala ᴅ/ʟ value is similar between the British and German records for both FAA and THAA ( Figure 5), but the range was larger for both German regions. Mean values were slightly lower for east Poland & Lithuania, but the range of ᴅ/ʟ values was also broader.

North of the Thuringian Basin, the nearby site of Neumark Nord II (Geiseltal - Central German Dry Area) has been attributed to the Eemian based on strong independent bio- and chronostratigraphic evidence, including a full Eemian pollen sequence and evidence for the Blake Event (0.12 Ma; Sier *et al.*, 2011; Sier *et al.*, 2015). Other sites in this region attributed to the Eemian on the basis of biostratigraphy include Burgtonna (SI; Kahlke, 1978; Meyrick & Maul, 2002; Zeißler, 1970), Taubach (SI; Bratlund, 1999; Brunnacker *et al.*, 1983; Götze, 1892; Kahlke, 1977; Müller, 1902; Steiner, 1977; Weiss, 1910; Zeißler, 1977; Ziegenhardt, 1962) and Weimar Parkhöhle (SI; Mania, 1984; Zeißler, 1962; Zeißler, 1967). Opercula from these sites produced similar but slightly lower values to Neumark Nord I ( Figure 4b; Penkman, 2010) and Neumark Nord II ( Sier *et al.*, 2011). The Thuringian Basin sites of Burgtonna, Taubach and Weimar Parkhöhle are the only sites within these four aminostratigraphies located higher than 200 m.a.s.l (metres above sea level). It is possible that being positioned at higher elevation in the heart of the Thuringian Basin may have resulted in exposure to different micro-climates, and consequently slightly different temperature histories to sites located at lower elevations neighbouring the Thuringian Basin, such as Neumark Nord I & II.

Sites attributed to the Eemian in the NE German Plain include Klein-Klütz-Höved profiles 1 and 2, Lichtenberg Li-BPa borehole horizons 1300–1325 cm and 1143–1163 cm, and horizons from the Stendal and Strausberg boreholes ( Figure 4a). All sites were correlated with Eemian pollen sequences (see SI). The two opercula-bearing horizons in the Lichtenberg Li-BPa borehole were correlated with high resolution palynological results ( Hein *et al.*, under review), which assigned the older layer to the Pre-temperate pollen zone E I/E1 and the upper horizon to the Late-temperate pollen zone E V/E6/7 ( Erd, 1970; Erd, 1973; Menke & Tynni, 1984). The age of these deposits was further constrained by 11 quartz OSL ages and 23 fading-corrected pulsed IR50 and pulsed pIRIR225 ages ( Hein *et al.*, under review; Rahimzadeh *et al.*, 2024). The Bayesian age-depth model places the deeper of the two horizons in the earliest part (ca. 126.5 to 128 ± 8.6 ka) and the shallower horizon in the later part of the interglacial (ca. 118 to 119 ± 8.2 ka). Similar ᴅ/ʟ values were observed for Lichtenberg when compared to the British MIS 5e and Neumark Nord II (see SI); ᴅ/ʟ values for all amino acids were higher for Li-BPa 1300–1325 cm and lower for Li-BPa 1143–1163 cm. This is consistent with the conclusions previously drawn regarding the age of this section of the Li-BPa borehole. ᴅ/ʟs from all other NE German Plain Eemian sites cluster around the samples from Lichtenberg, confirming they were formed in the same chronostratigraphic stage.

Three sites from east Poland (Puznówka, Słup 19 and Ławy) have been attributed to the Eemian on the basis of palynology (SI; Figure 4d). The ᴅ/ʟ values from these sites cover the same range as those from British MIS 5a/5c and MIS 5e material. This suggests that these are also likely to have been deposited in MIS 5, but have probably experienced slightly cooler temperatures on average compared to British opercula from the same chronostratigraphic stage. In addition, this indicates that this dataset covers a larger time period than demonstrated by the British record leading to a larger range of ᴅ/ʟ values.

The central Polish Eemian data spans the same range of ᴅ/ʟ values as the British MIS 5a/4 to MIS 7 data ( Figure 5; SI). There are two clusters of Ala ᴅ/ʟ values for opercula attributed to this stage ( Figure 4c); suggesting that the opercula within this group come from two different warm periods, probably two different interglacials. Opercula that yielded the lower extent of IcPD came from the sites of Kuźnica, Świątniki and Piła ( Figure 4c). Świątniki has been attributed to the Eemian on the basis of the molluscs present ( Alexandrowicz & Alexandrowicz, 2010). The *Bithynia* opercula occur in the same sedimentary horizon as the warm-loving mollusc *Belgrandia marginata* ( Michaud, 1831), signifying the climate optimum. ᴅ/ʟ values from this site are similar to the British MIS 5a-4, and the lowest ᴅ/ʟs from the Thuringian Basin Eemian. Piła, a site located further west in the Central Polish Lowlands, is attributed to the Eemian on the basis of the correlated pollen assemblages and molluscs ( Alexandrowicz & Alexandrowicz, 2010; Alexandrowicz *et al.*, 2024; Kuszell *et al.*, 2008). Kuźnica’s Eemian attribution was confirmed by IcPD.

The higher ᴅ/ʟ values come from the sites of Kopaszewko, Rogaczewo and Zbytki ( Figure 4c), and plot midway between the cluster of other sites attributed to the Eemian and the cluster attributed to the Mazovian/Holsteinian. This suggests that these sites either come from a warm episode between the Eemian and the Mazovian, or that conditions in Poland between these two interglacials did not reach temperatures required to accelerate the rate of IcPD sufficiently for substantial protein decomposition within the opercula to occur. The ᴅ/ʟs from all amino acids from these three sites are similar to both the British MIS 7 (e.g. Stanton Harcourt, Aveley, Ebbsfleet Channel, West Thurrock (Lion Pit); Penkman *et al.*, 2013, SI data and references therein) and German Saalian Complex (Dömnitz warm stage; Figure 5; SI Figs. S1–3) and higher than opercula material attributed to the early Eemian from Neumark Nord II (Geiseltal - Central German Dry Area, north of the Thuringian Basin; Sier *et al.*, 2011) and Lichtenberg (attributed to the Pre-temperate and Late-temperate phases of the Eemian).

All three sites were originally classified as Eemian due to the position of the deposits containing molluscs below Weichselian till ( Krzyszkowski & Winnicki, 1994) and the presence of tree pollen assemblages characteristic of the Eemian ( Kuszell, 1994a; Kuszell, 1994b); however, only Rogaczewo has a full interglacial pollen sequence. In all three profiles, the sediments in which molluscs were present are likely to represent an early, Pre-temperate phase of the interglacial. This is indicated by the presence of *Gyraulus laevis* ( Alder, 1838) (a cold-loving, pioneer taxon) towards the base of each profile, gradually disappearing towards the top. The presence of *G. laevis*, in addition to a lack of molluscan taxa with high ecological requirements, suggest sedimentation occurred during an early, Pre-temperate phase of an interglacial ( Alexandrowicz, 1994). This might indicate that the higher ᴅ/ʟ values represent the earliest part of the Eemian interglacial and lower ᴅ/ʟ values in this region represent the climate optimum/Late-temperate phases. However, the limited space between the ᴅ/ʟs associated with these sites and the Holsteinian cluster, and similarity to the ᴅ/ʟ values from Ludorf (NE Germain Plain, Saalian Complex/ Dömnitz warm stage), Klosterrohrbach and Karsdorf (north-east of the Thuringian Basin; Saalian Complex/ Dömnitz warm stage) and the early part of the British MIS 7 would suggest an older age attribution is more likely. Therefore, the IcPD results of this study suggests a reattribution of these opercula (Kopaszewko, Rogaczewo, and Zbytki) to an interglacial between the Eemian and Holsteinian. With no indications of reworking, this study therefore attributes them to the Saalian Complex.

***4.1.2 The Saalian complex***

The Saalian Complex describes deposits occurring after the Holsteinian Interglacial and prior to the Eemian Interglacial. The timing of this part of the Middle Pleistocene is still debated. Some have argued that it occurred between MIS 10 and MIS 6 ( Cohen & Gibbard, 2022; Lauer & Weiss, 2018) and includes both warm and cold stage deposits. However, some researchers previously proposed that this period of the Quaternary should be correlated with MIS 8 to MIS 6 (e.g. Geyh & Müller, 2007; Richter & Krbetschek, 2015). Four sites in this dataset have been previously attributed to the Saalian Complex on the basis of independent chronology ( Figure 4 & Figure 5).

One site, Ludorf from the NE German Plain ( Figure 4a), has been attributed to the early Saalian Complex/late Holsteinian due to the presence of *Azolla filiculoides* (water fern), which is not known in Europe after the Saalian Complex (e.g. Bertelsen, 1972). Other associated pollen is indicative of a transition, which was interpreted as the end of the Holsteinian Interglacial to the beginning of the Fuhne cold period (Fuhne A). The palynological data from these sediments is fragmentary, therefore a Dömnitz age cannot be ruled out. IcPD values from Ludorf were significantly lower than other Holsteinian horizons from the NE German Plain ( Figure 4a & Figure 5; SI), but at the higher range of ᴅ/ʟ values observed in Saalian Complex material from the Thuringian Basin and Poland. The average Ala ᴅ/ʟ for Ludorf lies within the higher range of MIS 7 Ala ᴅ/ʟ values from the British record. IcPD analysis therefore supports correlation with an interglacial period within the Saalian Complex for this site.

To the east of the Thuringian Basin, two sites: Karsdorf and Klosterrohrbach (Burgenlandkreis), have been attributed to the Saalian Complex based on the presence of *Corbicula fluminalis* ( Müller, 1774). *Corbicula* are known in NW Europe for all post-Elsterian interglacials with the exception of the Eemian ( Meijer & Preece, 2000). Deposits from Klosterrohrbach have been attributed to the Dömnitz warm period following pollen analysis (RPAZ 4a-b), which has been correlated with MIS 7 ( Endtmann *et al.*, 2024). Both sites yielded higher ᴅ/ʟ values than Eemian deposits from the Thuringian Basin, and lower values than samples attributed to the Holsteinian at Bilzingsleben ( Figure 4b & Figure 5; SI: Daniel & Frenzel, 2023; Hutson *et al.*, 2025; Stahlschmidt *et al.*, in prep). This corroborates their assignment to the Saalian Complex. As Karsdorf has yielded higher ᴅ/ʟ values than Klosterrohrbach, it is likely this is an older deposit, either from an earlier stage in MIS 7 or an earlier warm period in the Saalian Complex.

The Snaigupėlė Interglacial deposits at Valakampiai, Lithuania, are often attributed to MIS 7 (e.g. Gaigalas *et al.*, 2005; reviewed in Šeirienė & Bitinas, 2024), but this interglacial has also been attributed to MIS 5 ( Molodkov *et al.*, 2002). Snaigupėlė Interglacial deposits from other localities have also been correlated with MIS 7 (e.g. Gaigalas *et al.*, 2007; Kondratienė & Damušytė, 2009; Šeirienė *et al.*, 2019), MIS 5e (e.g. Baltrūnas *et al.*, 2015) and MIS 9 (e.g. Baltrūnas *et al.*, 2019; Šeirienė *et al.*, 2019). The ᴅ/ʟ values from this deposit are similar to the lower range of the MIS 7 ᴅ/ʟ values from the British record ( Figure 4d & Figure 5; SI), and are higher than ᴅ/ʟ values from Eemian deposits from the Podlasie Lowland. This supports the attribution of MIS 7 for the Snaigupėlė Interglacial at Valakampiai. MIS 7 deposits at Valakampiai yielded lower ᴅ/ʟ values than Saalian Complex sites from Germany. This could be due to Lithuania being on average colder than Germany since MIS 7 ( Gamisch, 2019; Timmerman *et al.*, 2022), or that material from the Snaigupėlė Interglacial is from a later part of this complex MIS than the Saalian Complex *Corbicula* gravels.

All samples attributed to the “Saalian Complex” in the German and Polish records fall within the range of MIS 7 in the British record (e.g. Stanton Harcourt; Aveley, Ebbsfleet Channel, West Thurrock (Lion Pit); Penkman *et al.*, 2013, SI data and references therein). This could suggest that all the Saalian Complex material in this dataset comes from MIS 7 (with the older material from the earliest substage MIS 7e, and the younger material from a later substage, MIS 7a/c). Alternatively, this could indicate that opercula deposited in MIS 9 and 7 in northern and central Germany and Poland were exposed to colder integrated burial temperatures than those from the British Isles, and as a result the German and Polish samples have a lower extent of IcPD and do not cover such a large range of ᴅ/ʟ values.

***4.1.3 Middle Pleistocene***

Two sites in this analysis, Höngeda and Zeuchfeld-Borntal, have been attributed to the Middle Pleistocene, with no further evidence to constrain these deposits to a narrower stage of the Quaternary (T. Meijer, pers. comm.). IcPD results from Höngeda suggest that this deposit dates to the early Eemian or late Saalian Complex. The extent of IcPD in the Zeuchfeld-Borntal material suggests this deposit dates to the early part of the Middle Pleistocene (e.g. the Cromerian Complex) or the latest part of the Early Pleistocene ( Figure 4b & Figure 5; SI Figs. S1–S3).

***4.1.4 Holsteinian***

The Holsteinian Interglacial succeeds the Elsterian, the latter correlated to MIS 12 ( Lauer & Weiss, 2018) but the precise timing of this period and its correlation with the MIS is still debated. The Holsteinian is commonly correlated with MIS 11c (e.g. Cohen & Gibbard, 2022; Koutsodendris *et al.*, 2012; Nitychoruk *et al.*, 2005; Sarnthein *et al.*, 1986), the British Hoxnian (e.g. Bridgland, 1994; Horne *et al.*, 2022; Rowe *et al.*, 1999), and the Polish Mazovian (e.g. Górecki *et al.*, 2022; Nitychoruk *et al.*, 2005; Szymanek, 2011; Figure 2). However, correlations of the type-site have been made to MIS 9 on the basis of ^230^Th/U dates from two peat layers ( Geyh & Müller, 2005; Geyh & Müller, 2007). The methods used to yield these ages have been questioned and new ^230^Th/U by Sierralta *et al.* (2017) of the para-stratotype in Mecklenburg-Vorpommern and Bossel suggest the original analyses were subjected to open system conditions ( Preece *et al.*, 2007). In Britain, it is now recognised that the “Hoxnian” pollen succession is present in more than one interglacial and sites with similar “Hoxnian” pollen have since been correlated with MIS 9 (e.g. Cudmore Grove; Penkman *et al.*, 2013, SI data and references therein) and 11 (e.g. Ashton *et al.*, 2008; Bridgland, 1994; Bridgland *et al.*, 2001; Candy *et al.*, 2021; Preece *et al.*, 2007; Roe *et al.*, 2009; Roe *et al.*, 2011; Rowe *et al.*, 1999; Scourse *et al.*, 1999). In this data analysis, “Holsteinian” sites are considered together. Sedimentary horizons correlated with Holsteinian/Mazovian pollen successions have been subdivided into pre-, early-, late- and post-temperate phases after Turner and West (1968) (see SI 7.1: Table S3 and SI 7.3). For sites attributed to either the British Hoxnian, German Holsteinian and Polish Mazovian, the mean IcPD decreases from west to east, along the gradient of increasing climate continentality ( Figure 5; e.g. Mikolaskova, 2009; Stonevicius *et al.*, 2018).

Opercula from the NE German Plain have been previously attributed to the Holsteinian due to the occurrence of Holsteinian pollen sequences and the presence of the “Paludinenschichten” (see SI; sediments containing the now extinct aquatic gastropod *Viviparus diluvianus* (e.g. Schmierer, 1922; Steusloff, 1953)). This species is known from Tiglian and Holsteinian deposits in the Netherlands (e.g. Boettger, 1955; Meijer, 1990; reviewed in Szymanek, 2011). The range of ᴅ/ʟ values in amino acids presented ( Figure 4a & Figure 5; SI) for the NE German Plain Holsteinian material covers the same range as MIS 9 (e.g. Cudmore Grove, Barling, Belhus Park, Grays, Purfleet; Penkman *et al.*, 2013: SI data and references therein), MIS 11 (e.g. Hoxne, Barnham, Clacton-on-Sea, Mark’s Tey, Swanscombe; Penkman *et al.*, 2013: SI data and references therein) and late Cromerian (Waverley Wood; Penkman *et al.*, 2013, SI data and references therein) material from the British record. The ᴅ/ʟ values are normally distributed. While this might be because the “Holsteinian” represents a single interglacial, interestingly when the data from MIS 9 and MIS 11 “Hoxnian” sites from Britain are combined, this also shows a normal distribution, so the presence of two interglacials within the dataset would not have been apparent from the IcPD data alone. Based on IcPD, we therefore cannot rule out that “Holsteinian” material may be associated with two interglacials. There is potential for regional pollen assemblages to resolve interglacial stages where resolution is not possible using IcPD; this is investigated in Section 4.2.3.

The sole site from the Thuringian Basin attributed to the Holsteinian is the archaeological site of Bilzingsleben ( Eissmann, 1994; Mania, 1995; Unger & Kahlke, 1995; reviewed in Pasda, 2012). Opercula from the sand layer below the travertine were analysed by IcPD. Previous work to date these deposits have involved U-series dating of the overlying travertine/tufa deposits ( Brunnacker *et al.*, 1983; Harmon *et al.*, 1980; Mallick, 2001; Schwarcz *et al.*, 1988) and ESR of overlying tufa and rhinoceros tooth enamel from the tufa sands ( Schwarcz *et al.*, 1988), correlating these deposits with MIS 7 to greater than MIS 9. Others have correlated Bilzingsleben to MIS 11 ( Bridgland *et al.*, 2004; Jöris & Baales, 2003; Steguweit, 2003). More recent work to constrain the age and environmental context of the site includes analysis of the mammalian biostratigraphy ( Müller & Pasda, 2011), ostracods ( Daniel & Frenzel, 2023) and pIRIR _290_ and IR-RF luminescence dating ( Stahlschmidt *et al.*, in prep). The extent of IcPD from the “Holsteinian” Bilzingsleben opercula were similar to the ᴅ/ʟ values observed for the British MIS 11 material and the “Paludinenbank” deposits at Berlin ( Figure 4b & Figure 5), supporting correlation of these deposits to the Holsteinian Interglacial.

The Polish material has been attributed to MIS 11 due to association of the opercula to Holsteinian pollen sequences and position above the Sanian 2/ Elsterian tills (SI). One age reassignment has been made by IcPD for the Polish material ( Figure 4c). The central Poland site of Wildno was originally assigned to MIS 3 on the basis of a ^14^C date on shell detritus (34,159 ± 906 cal BP; Dzierżek & Szymanek, 2013). However, IcPD analysis suggested this site should be reassigned to MIS 11. Based on the geomorphological situation, the most probable explanation is that the MIS 11-aged sediments were redeposited by the ice sheet (as glacially transported sediment masses) or, although less probable, that their high position results from glacio-tectonic deformations. Alternatively, if the younger ^14^C dates are considered to be reliable, we can also expect some mixing of older and younger shell material due to fluvial processes.

The ᴅ/ʟ values for the Polish Mazovian are lower than the German Holsteinian and British MIS 11 and 9 deposits ( Figure 4 & Figure 5). This further suggests that the integrated temperature has been colder in Poland compared to the other regions. This is to be expected if trends in temperature follow modern trends in MAT, seasonality and continentality discussed in Section 2.1.

***4.1.5 Pre-Holsteinian***

Material older than the Holsteinian is associated with the Elsterian, the locally defined the Arternian (Thuringian Basin), and the Augustovian (=Podlasian; NE Poland) Interglacials. The Elsterian is the glacial stage preceding the Holsteinian Interglacial within Northwestern Europe. The Arternian Interglacial is the term for a regional interglacial deposit within the Thuringian Basin, attributed previously to MIS 29-21 by various independent age evidence ( Maul *et al.*, 2013). The oldest stage presented in these aminostratigraphies is the regional Augustovian/Podlasian Interglacial from north-east Poland, which has been correlated with the Dutch Bavel Interglacial, and is believed to have occurred at the start of the Jaramillo subchron (~1.1 Ma; Ber, 2006).

The deposits that have been attributed to the Elsterian are Uichteritz and Jena, from the Burgenlandkreis and Thuringian Basin respectively. The opercula from the Uichteritz gravel pit (Burgenlandkreis) were located near the contact between the early Elsterian Lower Gravel Unit (without Nordic till) and late Elsterian Middle Gravel Unit ( Meng & Wansa, 2005). The IcPD is similar to the highest Holsteinian values from the NE German Plain, and the lowest values from the British Late Cromerian material ( Figure 4b & Figure 5; Penkman *et al.*, 2011; Penkman *et al.*, 2013). Consequently, IcPD supports a late Elsterian age for the Uichteritz material. The other site suspected to be Elsterian is Jena, although the chronology of this deposit is uncertain (Meng, in pers. comms to E. Nelson). ᴅ/ʟ values for this site are significantly higher than that of Uichteritz, and more similar to ᴅ/ʟ values from the British early Cromerian sites and the Arternian Interglacial from Voigtstedt, which has been correlated with MIS 29-21 (~1-0.81 Ma; Maul *et al.*, 2013). Therefore, IcPD suggests that the Jena opercula were precipitated in either the earliest stages of the Middle Pleistocene or the late Early Pleistocene.

The Augustovian Interglacial material comes from the site of Komorniki in the Augustów Plain. This interglacial was correlated with the Bavelian Complex or Cromer I on the grounds of palynology and geological context (Ber, 1996; reviewed in Ber, 2006; Ber *et al.*, 1998; Janczyk-Kopikowa, 1996; Nitychoruk *et al.*, 2000; Winter, 2001). As such it should be older than the Elsterian deposits and the British Cromerian material, and of a similar age to the Arternian Interglacial at Voigtstedt. The ᴅ/ʟ values from Komorniki are higher than Holsteinian aged material from the east Poland and Lithuanian sites, supporting the conclusion that the Komorniki material is older ( Figure 4d & Figure 5). However, this material is less racemised than both the British Cromerian opercula and the Arternian Interglacial material from the Thuringian Basin. Given the strong independent evidence of age, the IcPD results suggest that the integrated burial temperatures in the north-east of Poland have been significantly colder.

***4.1.6 Proposed changes to age attribution based on IcPD***

Following the IcPD results from each individual regional aminostratigraphic framework, and comparison to the British record, recommendations for a new age attribution of seven sites have been made and summarised in Table 2.

**Table 2.** A summary of the sites where age has been reassigned following IcPD analysis.

Site	Horizon	Original attributions	New attributions based on IcPD
Wildno	5–3 m	MIS 3	MIS 11
Rogaczewo	Eemian horizon	Eemian	Saalian Complex/ MIS 7
Kopaszewko	Eemian horizon	Eemian	Saalian Complex/ MIS 7
Zbytki	Eemian horizon	Eemian	Saalian Complex/ MIS 7
Jena	1020/1/1	Elsterian	Early Middle Pleistocene/ late Early Pleistocene
Höngeda	MTG-447	Middle Pleistocene	Early Eemian/ late Saalian Complex
Zeuchfeld-Borntal	Schicht 7 - NITG-445	Middle Pleistocene	Early Middle Pleistocene/ late Early Pleistocene

### 4.2 IcPD patterns in pollen successions

**Figure 6.**
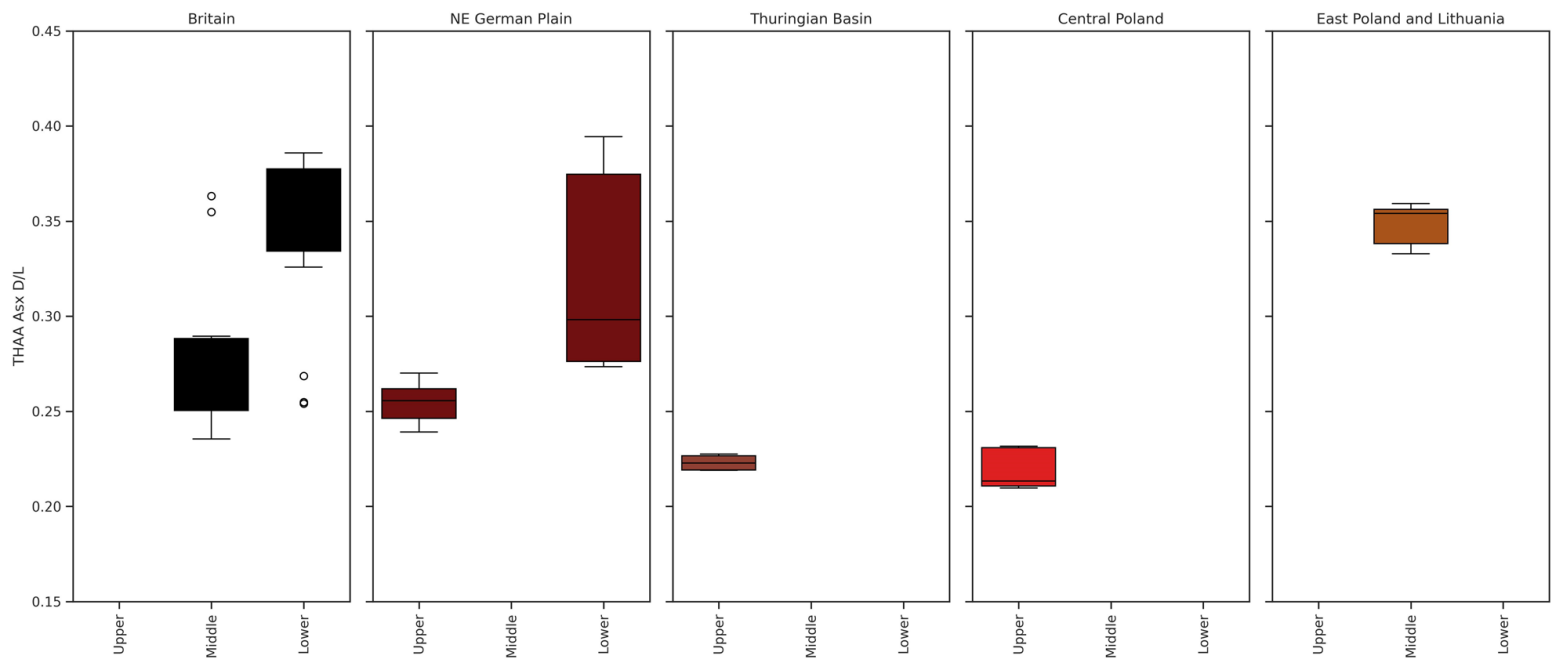
The extent of THAA Asx ᴅ/ʟ for each Holocene substage for each region. A similar extent of IcPD is evident for all regions for material of this age, with higher D/Ls in earlier parts of the pollen successions where samples are available. Substages of the Holocene are described in SI Table S1.

The majority of sites in this study are attributed to the Holocene, Eemian or Holsteinian interglacial periods. A large proportion of these sites yielded opercula correlated with an interglacial pollen zone (PZ), which given the pattern of pollen successions is indicative of the timing of these horizons within the interglacial (e.g. Cheddadi *et al.*, 1998; Erd, 1970; Erd, 1973; Hermsdorf & Strahl, 2008; Menke & Tynni, 1984; Phillips, 1974; Turner, 2002; Turner & West, 1968; West, 1956; West, 1964; Zagwijn & de Jong, 1984; Zagwijn, 1996). To determine whether IcPD allows temporal resolution within these interglacial successions, and explore any differences observed between the Northern European Plain and British aminostratigraphies, this study compares the IcPD results to available PZ data. These are not uniform across different regions for each interglacial and the timing of each stage may differ across continental Europe depending on regional responses to the changing climate (e.g. Brewer *et al.*, 2008; Sánchez Goñi *et al.*, 2005). In addition, a marine transgression into the NE German Plain occurred in both the Eemian (e.g. Meng *et al.*, 2022) and Holsteinian (e.g. Börner *et al.*, 2019), resulting in some PZs missing in the pollen succession of some sites (e.g. Menzel-Harloff & Meng, 2015). Therefore, to enable cross-comparison between regional pollen stratigraphies, each interglacial has been subdivided into a Pre-temperate, temperate (including the Early-temperate and Late-temperate), and post-temperate phase (SI: Tables S1–S3; SI 1.5) which describe similar and recurring sub-periods of vegetation development within an interglacial ( Turner & West, 1968); the differences in extent of IcPD between these phases of each interglacial are demonstrated in Figure 6– Figure 8.

***4.2.1 Holocene***

The Holocene (~11.7 ka to present) is the current interglacial. Comparisons of the Lower (Greenlandian; 11.7-8.2 ka), Middle (Northgrippian; 8.2-4.2 ka), and Upper (Meghalayan, 4.2 ka to present) Holocene material have been made using the amino acid Asx ( Figure 6), as it is a faster racemising amino acid and provides better temporal resolution for material of this age. The mean THAA Asx ᴅ/ʟ values for the Thuringian Basin and NE German Plain are similar to the THAA Asx ᴅ/ʟ values for the British Holocene ( Figure 6). In general, IcPD is highest at the start of the interglacial and lowest at the end, with similar extents of racemisation observed between substages.

The only Holocene-aged opercula from east Poland & Lithuania come from Skołoszów, the most southerly site in this aminostratigraphy (~300 km south of the nearest sites in the Podlasie Lowland and Western Polesie). The sedimentary horizon containing the opercula is associated with Atlantic stage pollen, placing this in the Middle Holocene. The extent of IcPD for the Skołoszów deposits is similar to levels of IcPD to the Lower Holocene age deposits from Britain and NE German Plain but higher than the Middle Holocene deposits from the British record (7.64 to 5 ka cal BP; e.g. Preece, 1998). This implies that either Skołoszów is older than the Middle Holocene, or it has been exposed to warmer temperatures throughout its burial history compared to the British Middle Holocene opercula. It is also possible that the level of resolution achieved by IcPD analysis is not sufficient to resolve the Middle and Lower Holocene. Further IcPD analyses of Holocene deposits across northern Europe are required before this can be determined.

In summary, it is possible to distinguish between Early and Late Holocene deposits with IcPD throughout northern Europe, but temporal resolution between the Early and Middle Holocene may not be possible.

***4.2.2 Eemian***

Eemian PZs have been defined across northern Europe and cover an approximately ~18,000-11,000-year period (e.g. Cohen & Gibbard, 2022; Lauterbach *et al.*, 2024; Müller, 1974a; Sánchez Goñi, 2007). The subdivision of the PZs used here is described in SI 7.1: Table S2 and SI 7.3. Compared to the Holsteinian, the Eemian is characterised by a very uniform, and relatively rapid succession of pollen of temperate wooden taxa, with a Late-temperate expansion of *Carpinus* (e.g. Turner, 2000; Tzedakis, 2007).

**Figure 7.**
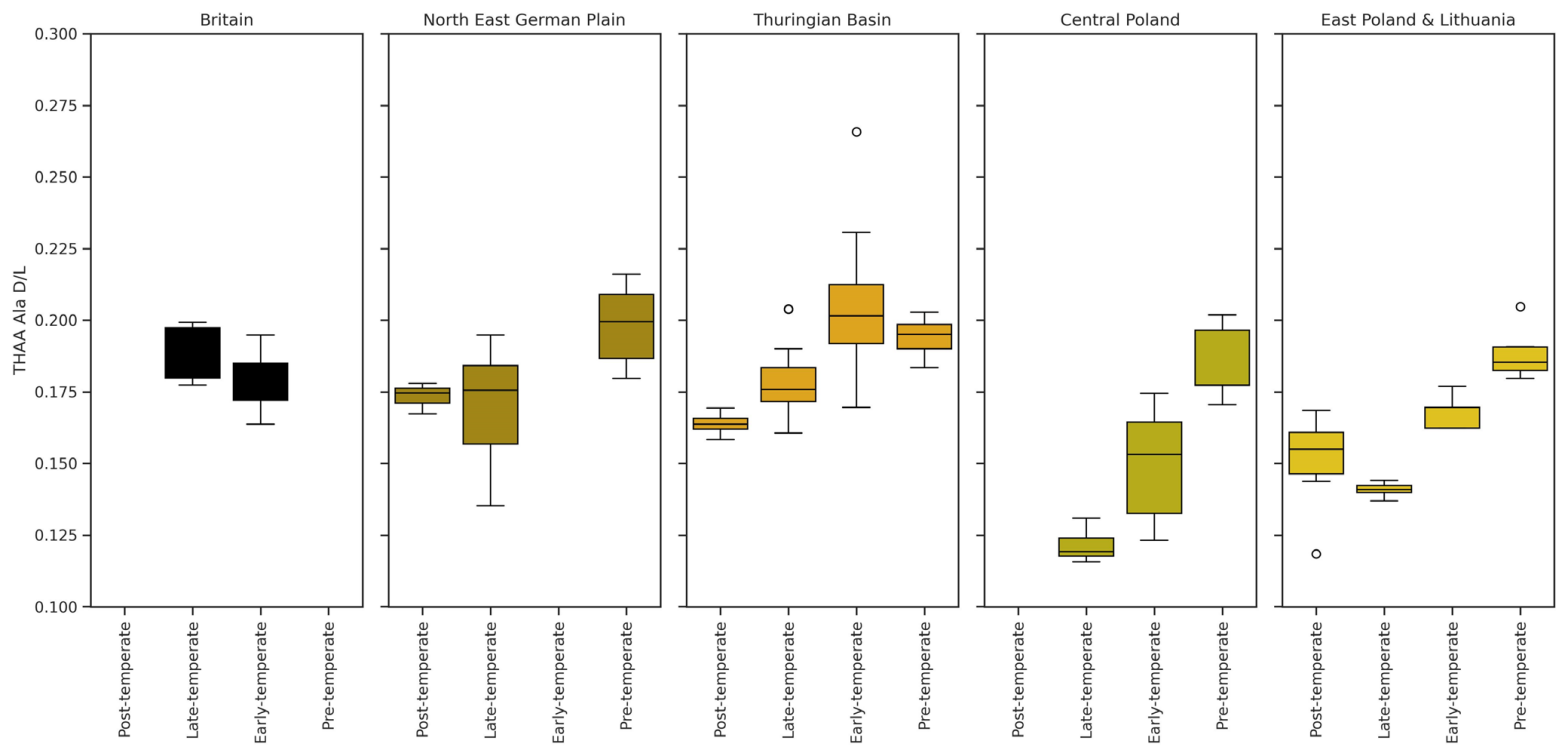
The extent of THAA Ala ᴅ/ʟ for each Eemian substage for each region. Eemian biostratigraphic zones vs. THAA Ala ᴅ/ʟs. ᴅ/ʟs are higher for the earlier pollen stages and lower for the later pollen stages within this interglacial. The range of THAA Ala ᴅ/ʟ values is lower for Central and East Poland & Lithuania compared to the other regional aminostratigraphies.

Several of the horizons in this study can be correlated with a particular pollen zone within the Eemian ( Figure 7). In general, for the eastern North European Plain regional records, THAA Ala ᴅ/ʟ are highest in the earliest pollen stages and lowest in the latest pollen stages. Horizons from the British record only represent temperate conditions, and so we cannot test for any difference between the start and end of the interglacial. The range of ᴅ/ʟ values for the Polish Eemian tends to be lower than the German Eemian and British MIS 5e material, suggesting a slight temperature difference between the two areas. Differences in the extent of racemisation between MIS 5e deposits due to MAT difference along a latitudinal gradient has been observed along the Pacific Coast of North America ( Wehmiller, 1982). In that region the 10° difference in latitude results in a ~10°C increase (~13.5 to 22°C) in present day MAT. The range of leucine ᴅ/ʟ values observed from MIS 5e deposits across the Pacific Coast region studied was ~0.4–0.7, rising in correlation with MAT. The difference in MAT is not so extreme between Britain and the North-East European Plain - in addition MATs are cooler than along the Pacific coast - therefore, the difference in the ᴅ/ʟ values is smaller.

IcPD can achieve better temporal resolution between the pre-, early and Late-temperate zones of the interglacial for the Polish material when compared to the German regional records. A possible explanation for this is that there is a larger difference in temperature between the peak of the Eemian Interglacial and the onset of the preceding cold stage in Poland than there was in Germany and Britain. This demonstrates that it is possible to distinguish between the beginning and end of the Eemian Interglacial in the regions investigated here, and possibly to distinguish between individual pollen subzones in Poland. Resolution was also achieved between the Pre-temperate and Late-temperate zones associated with opercula from two sedimentary horizons in the Lichtenberg Li-BPa core. Therefore, the degree of temporal resolution achievable with IcPD will vary depending on the climate associated with each regional aminostratigraphic framework.

***4.2.3 Holsteinian***

The Holsteinian and Hoxnian interglacials are characterised by their pollen sequence ( Erd, 1970; Menke & Tynni, 1984; Selle, 1962; Turner, 1970; West, 1956; SI Table S3), with the dominance of *Taxus* and Quercetum mixtum elements in the Early-temperate phase, followed by the Late-temperate phase: *Abies, Carpinus* and *Buxus* dominated zone. In addition, *Fagus*, *Abies, Celtis* and *Pterocarya* are present in the final phase of the interglacial (e.g. Erd, 1970; Erd, 1973; Müller, 1974b; Turner & West, 1968). Evidence of the predominance of *Abies* during the climate optimum of both MIS 11 and MIS 9 is observed in continuous pollen sequences in the French record (e.g. Reille *et al.*, 2000). In the German Holsteinian, *Abies* occurs together with *Carpinus,* however, in the Reinsdorf Interglacial pollen sequence (correlated with MIS 9) *Carpinus* dominates before *Abies*, the frequency of which varies depending on the location ( Strahl, 2019; Urban, 1995; Urban *et al.*, 2023; Wansa *et al.*, 2022), suggesting the Holsteinian and Reinsdorf were not contemporaneous.

**Figure 8.**
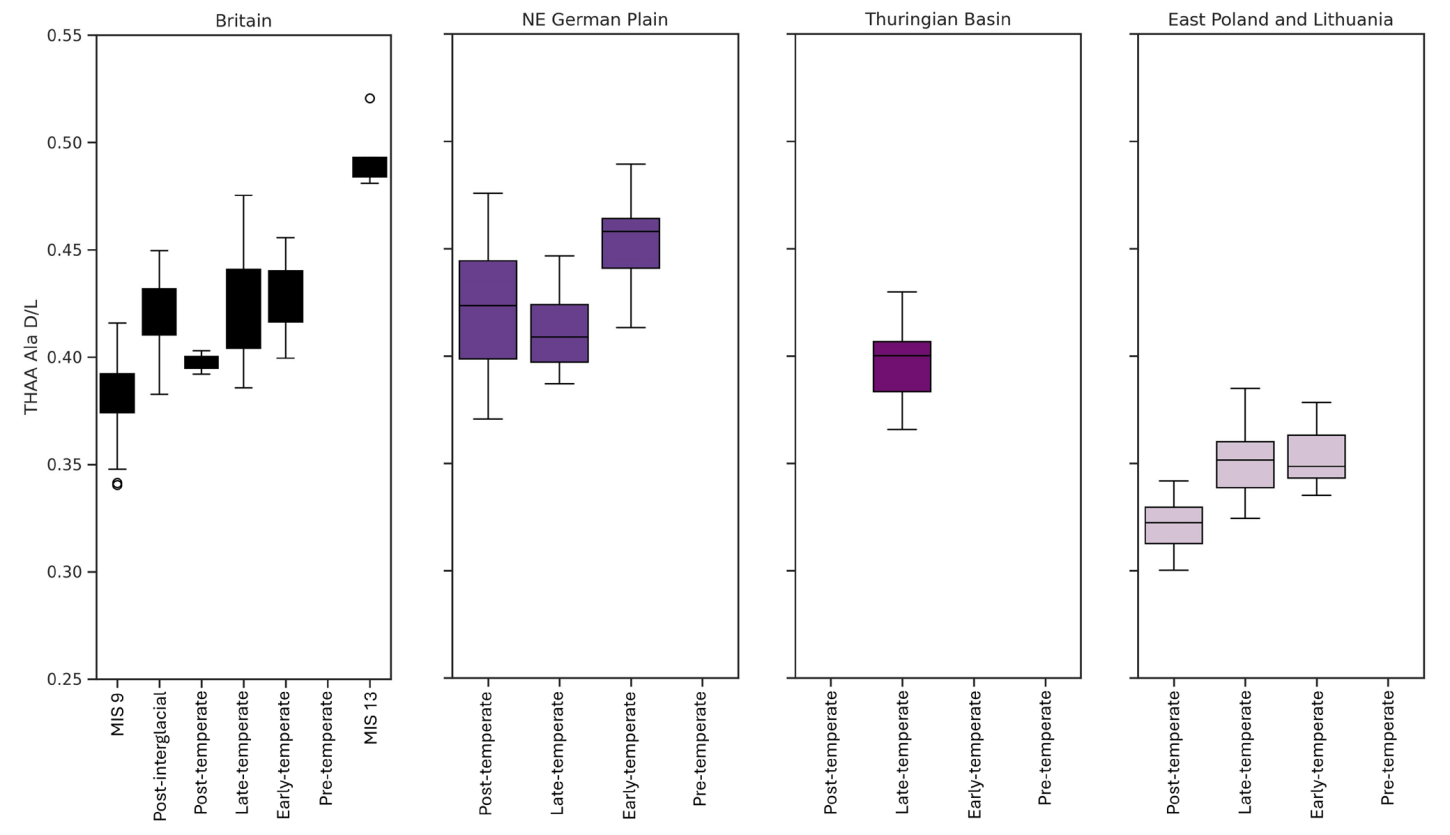
Holsteinian biostratigraphic zones vs. THAA Ala ᴅ/ʟs. Generally, ᴅ/ʟs are higher for the Pre-temperate phase compared to the Post-temperate phase of the Holsteinian but there is less resolution between pollen zones when compared to the Eemian. The range of IcPD values decrease with increasing continentality from west to east.

In general, ᴅ/ʟ values are slightly higher at the start of the interglacial and lower at the end of the Holsteinian Interglacial for each region ( Figure 8), but not such a substantial difference as is seen in the Eemian record ( Section 2.2), which suggests IcPD struggles to yield such clear resolution for material of this age. Reduced temporal resolution for this point in time is also observed in the British aminostratigraphy, where opercula dated to late MIS 11 (e.g. Mark’s Tey; Trimingham, Elveden; Penkman *et al.*, 2011; Penkman *et al.*, 2013 and references therein) cannot be resolved from early MIS 9 specimens (e.g. Shoeburyness, Purfleet, Grays; Penkman *et al.*, 2011; Penkman *et al.*, 2013: Supplementary Data and references therein). It is possible that this could be due the original stratigraphic attribution being incorrect. However, it is more likely due to two aspects: (i) the slowing/pausing of IcPD in the cold stages means that the end of one interglacial is hard to distinguish from the beginning of the next ( Bates, 1993; Miller *et al.*, 1999); and (ii) the initial racemisation rates are relatively fast, but as the ᴅ/ʟ values approach equilibrium, the curve flattens. Both of these factors means that the ability to discriminate between opercula associated with consecutive warm stages decreases as the protein becomes more degraded (e.g. Goodfriend, 1991; Kvenvolden *et al.*, 1979; Kvenvolden *et al.*, 1981; Wehmiller & Belknap, 1978). As a result, temporal resolution is poorer in older material.

The difference in regional mean ᴅ/ʟ values for the entire interglacial is greater than that observed for the Eemian IcPD results ( Section 4.2.2). This is consistent with the longer exposure to the different burial temperatures having a greater effect on IcPD. This also aligns with observations for deeply buried material (below 80 m depth) exposed to different burial temperatures ( Nelson *et al.*, 2024).

There is a larger than expected range of ᴅ/ʟ values for the Holsteinian sites from the NE German Plain record. To determine why that is, the ᴅ/ʟ values for each vegetation sub-period have been compared to values from MIS 11 and MIS 9 in the British record ( Figure 8). In the British record there appears to be little temporal resolution between the different pollen stages within MIS 11, but on average ᴅ/ʟ values from MIS 11 sites are higher than those of MIS 9. In the NE German Plain record, the highest ᴅ/ʟ values come from the sites of Vorketzin and Gorlosen. The Vorketzin opercula were associated with pollen indicative of a transitional phase (PZ 1 (birch period) and early PZ 2 (pine-birch-spruce-alder period)), either at the beginning or end of a warm period (Meng, in pers. comms to E. Nelson; Strahl, in pers. comms to E. Nelson). IcPD results suggest that the beginning of the interglacial, when temperatures were still relatively cold, is more likely. Gorlosen is also associated with pollen assemblages from the Pre-temperate phase of the Holsteinian (PZ 1 and 2 *sensu* Erd, 1973). These opercula have similar ᴅ/ʟ values to those for MIS 13 in the British record (Waverley Wood; Penkman *et al.*, 2013; Shotton *et al.*, 1993). This suggests that it may not be possible to resolve the earliest phase of the Holsteinian from the preceding interglacial. However, as there are no deposits associated with MIS 13 from the NE German Plain, this cannot be confirmed.

IcPD for the Pre-temperate zone is higher than the temperate phase of the Holsteinian. The NE German Plain Early-temperate and Post-temperate phases ᴅ/ʟ values are similar to that of the British MIS 11 and temperate Holsteinian material from the Thuringian Basin (Bilzingsleben; Daniel & Frenzel, 2023; Erd, 1997; Schreve *et al.*, 2002; Stahlschmidt *et al.*, in prep).

There is no Holsteinian (Mazovian) site from central Poland associated with pollen, so no conclusion can be made on the temporal resolution achievable by IcPD in this region for this interglacial. For east Poland & Lithuania, the mean ᴅ/ʟ values for the beginning of the interglacial are higher than the final stages of the interglacial. However, there is a large range of ᴅ/ʟ values for the Post-temperate substage in east Poland & Lithuania, which may be due to reworked opercula within the Szymanowo 0.65 m horizon. On the basis of this limited dataset, it is not possible to temporally resolve each substage with IcPD. Generally, ᴅ/ʟ values for each vegetation sub-stage is lower in the Polish material compared to the NE German Plain, and lower than the range observed in the British material for most equivalent stages. This may be due to the lower extent of IcPD in this region, but may also hint that each warm stage lasted a shorter period of time than in Britain and Germany.

### 4.3 The possible causes of systematic differences in IcPD across the eastern North European Plain

All four aminostratigraphies were produced from opercula where the closed system of the intra-crystalline protein fraction was retained (e.g. Nelson *et al.*, 2024; Nelson *et al.*, 2025a; Penkman *et al.*, 2011; Penkman *et al.*, 2013; Penkman *et al.*, 2024; Preece *et al.*, 2020; Tesakov *et al.*, 2020). Therefore, the extent of IcPD will have been due to the time since biomineralisation of the operculum and its integrated burial temperature history. In this study, the extent of IcPD for opercula attributed to broadly concurrent regional chronological stages of the Quaternary were compared (e.g. Cohen & Gibbard, 2022; Szymanek & Julien, 2018). However, the sites included in the regional aminostratigraphies discussed here are located across a wide range of northern Europe (from Britain to Lithuania), with different research traditions and approaches involved in determining age attributions and correlations. Although much progress has been made in this field (e.g. Böse *et al.*, 2012; Cohen & Gibbard, 2019; Cohen & Gibbard, 2022; Lauer & Weiss, 2018; Marks, 2023; Maul *et al.*, 2013; Sier *et al.*, 2011), age attributions may differ between regions, additionally some of the variability between individual frameworks may be because climate change has occurred asynchronously across Europe. Here, we have employed the current understanding of regional Quaternary chronostratigraphy correlations, but it is possible that future work may change some age attributions, which may explain some of the differences between and internal variability of the extent of IcPD from the five regional aminostratigraphies discussed here.

There is a longitudinal trend in the extent of IcPD between the five regional aminostratigraphies, with generally higher IcPD for more westerly sites and lower for more easterly sites. This difference accumulates with age of the material. In northern Europe, the climate transitions from an oceanic temperate climate in Britain to an increasingly continental climate with distance from the Atlantic Ocean, North and Baltic Seas (e.g. Mikolaskova, 2009; Stonevicius *et al.*, 2018). It is likely that a marine-continental gradient has occurred throughout the Quaternary, although due to changes in sea level (e.g. Barlow *et al.*, 2017; Gibbard & Knudsen, 2024; Meng *et al.*, 2022; Streif, 2004) and precipitation patterns (e.g. Koutsodendris *et al.*, 2012; Kühl *et al.*, 2007) this gradient will have varied over time. At present, the increasing continentality from west to east in northern Europe leads to more severe, colder winters but drier and slightly warmer summers in Poland compared to Britain ( Table 1; Figure 3). In addition, average temperatures drop below freezing for longer periods of time in more easterly areas compared to Britain, when it is likely that the rate of racemisation slows or pauses entirely ( Miller *et al.*, 2000). Should a similar trend in continentality have persisted throughout the Quaternary, where north-east Europe was exposed to cold conditions for longer periods of time (e.g. Osman *et al.*, 2021), this may explain why lower levels of IcPD are observed in Poland when compared to Germany and the UK. Changes in the extent of racemisation due to changes in climate across continental areas were observed previously from the Pacific coast of North America by Wehmiller ( 1982; 1984). The extent of racemisation in leucine between equivalent aged sites (dated to ~120 ka by U/Th dating of corals; Kennedy *et al.*, 1982) ranged from ~0.4-0.7, rising with MAT (ranging between ~13.5 to 22°C). This demonstrates that differences in surface temperatures can have a significant impact of protein decomposition.

In northeastern Poland, deep-seated relict permafrost (at least 93 m thick) still exists below a depth of 357 m ( Szewczyk & Nawrocki, 2011; ~50 km north of Komorniki, Augustów Plain). This indicates that the remnants of the Last Glacial Maximum can still impact the thermal regime of the outer zone of the Earth’s crust in areas where very low heat flow density occurs due to low radioactivity. The intensity, thickness and persistence of permafrost following each glacial stage will have played an important role in the burial temperatures that opercula were exposed to. Reconstructing permafrost is challenging, but this evidence of relict permafrost due to low heat flow density in north-east Poland may also account for the lower extents of IcPD observed in the east Poland aminostratigraphy.

Future work should explore the effects of different variables that influence the integrated burial temperature, and therefore the extent of burial temperatures on IcPD. This will assist with the cross-correlation of different regional IcPD frameworks, helping to link these together in a broader European aminostratigraphic framework.

## 5 Conclusions

In this study, four new regional aminostratigraphic frameworks have been presented for the NE German Plain, Thuringian Basin, central and east Poland & Lithuania, expanding the range of the IcPD dating method to new geographical areas. All aminostratigraphies show that IcPD is able to provide useful temporal resolution, with the potential to extend beyond 1 Ma. Central Poland includes material from the Miocene and demonstrates that a closed system can be retained in opercula of this age. IcPD analysis has constrained the age of several sites with poor chronological control, and suggests that the previous age attributions of seven sites need to be revised and/or refined to a smaller time window within the Quaternary.

Correlation of opercula with regional pollen assemblages for interglacials from the last 500 ka has shown that in general, IcPD can distinguish between the beginning and end of an interglacial for the Holocene, Eemian and Holsteinian interglacials, but not individual pollen zones. Temporal resolution within an interglacial was higher for the Holocene and Eemian compared to the Holsteinian, likely due to decreasing rates in IcPD in older material (e.g. Goodfriend, 1991; Kvenvolden *et al.*, 1979; Kvenvolden *et al.*, 1981; Wehmiller & Belknap, 1978).

Systematic differences in IcPD were observed between sites assumed to be similar in age for the Polish Mazovian, German Holsteinian and British Hoxnian (typically attributed to MIS 11). In general, IcPD was lower for Polish material, whereas levels of IcPD within the two German regions were more similar to that observed for MIS 11 and MIS 9 sites in Britain. This suggests that central and east Poland have been on average colder throughout the Quaternary compared to the German regions and Britain, which meets expectations if trends in MAT and seasonality across northern continental Europe followed those observed today (e.g. Hersbach *et al.*, 2020). We therefore highlight the importance of developing regional aminostratigraphies (as demonstrated here), so that further independent evidence of age in these regional relative chronologies can be tied together. The range of IcPD values for the Holsteinian in the NE German Plain was greater than observed for both the British MIS 11 and MIS 9 sites. Additional evidence of age is required to confirm correlations of the Holsteinian with the MIS record.

This study has also demonstrated how differences in climate regimes over continental land masses at similar latitudes (51–53°N) can result in systematic differences in IcPD, which increases with increasing age of material. This is likely a result of increasing degree of continentality from west to east, potentially resulting in the persistence of colder conditions for longer in more easterly material. Future work should attempt to disentangle the relationship between integrated burial temperatures as a result of varying climate, with the aim of being able to correct for temperature differences. This will allow for cross-correlation of IcPD dating frameworks across northern Europe.

## 7 Ethics and consent

Ethical approval and consent were not required.

## Data Availability

The underlying data has been deposited in the Zenodo Repository: https://doi.org/10.5281/zenodo.17647957 ( Nelson *et al.*, 2025b) This project contains the following underlying data: **Sample Information Table:** Context of the opercula used in this study. Including lab information, correlated independent chronology, and pollen zones. **Supplementary information:** Pollen zones: description of pollen zonation schemes used for opercula deposits IcPD analysis: extended methodology Supplementary Figures **Supplementary data file:** Sheet 1 – Eastern North European Plain IcPD data, Sheet 2 – pollen zones correlated with opercula samples where both types of fossils were present in a sedimentary horizon. **Code availability:** Python scripts used for the data analysis within this work can be found at: 10.5281/zenodo.15800604. [ eNEP_IcPD_DataAnalysis.ipynb] Nelson (2025). eNEP_IcPD_DataAnalysis. [Python Script]. In Quaternary aminostratigraphies for the eastern North European Plain. Zenodo. 10.5281/zenodo.15800604 Data are available under the terms of the Creative Commons Zero "No rights reserved" data waiver (CC01.0 Public domain dedication). The British aminostratigraphy data and site information has been sourced from : Penkman, K.E.H., Preece, R.C., Bridgland, D.R., Keen, D.H., Meijer, T., Parfitt, S.A., White, T.S., Collins, M.J., 2011. A chronological framework for the British Quaternary based on Bithynia opercula. Nature 476, 446–449. doi: 10.1038/nature10305: **Supplementary Data 1–2** Penkman, K.E.H., Preece, R.C., Bridgland, D.R., Keen, D.H., Meijer, T., Parfitt, S.A., White, T.S., Collins, M.J., 2013. An aminostratigraphy for the British Quaternary based on Bithynia opercula. Quaternary Science Reviews 61, 111–134. doi: 10.1016/j.quascirev.2012.10.046: **Supplementary Material** Data are available under the terms of the Creative Commons Zero "No rights reserved" data waiver (CC01.0 Public domain dedication). Copyright: © 2025 Nelson E.F. *et al*. This is an open access work distributed under the terms of the Creative Commons Attribution License, which permits unrestricted use, distribution, and reproduction in any medium, provided the original work is properly cited.
