# Supplementary material for: Quaternary aminostratigraphies for the eastern North European Plain
Source: Open Res Eur. 2026 Jun 17;5:396. Originally published 2025 Dec 22. [Version 2] doi: 10.12688/openreseurope.21815.2 (PMC12811728; doi:10.12688/openreseurope.21815.2)
Supplement: Peer review history

# Reviewer report: Reviewer response for version 1

**DOI:** 10.21956/openreseurope.23597.r66952

**Published:** 2026-01-16

**Authors:** Ian Candy

**Affiliations:** 1 Royal Holloway, University of London, Egham, UK

The paper by Nelson et al. presents an extensive and original dataset of Amino Acid Racemisation (AAR) analysis on the calcitic opercula from *Bithynia* *tentaculata* sampled from a range of Pleistocene sites across central and eastern Europe. The prevalence of opercula within temperate deposits in this region means that the AAR analysis of such materials from a range of Pleistocene deposits provides the potential to correlate and/or subdivide deposits on the basis of their relative age. This has been done successfully for interglacial/interstadial deposits within the British Isles. The problem/scientific issue that this study hopes to address is an important one as the age and correlation of interglacial deposits in central/eastern Europe has been a scientific question that has been asked for most of the 20 ^th^ Century and, in many instances, is still being debated. The implications of this unresolved issue are far reaching, particularly in the study of Palaeolithic archaeology and human evolution. Many of the deposits discussed here, as is alluded to by the authors, contain evidence for early humans but the significance of this evidence requires a robust chronology/stratigraphy within which the archaeology can be placed. Without this the timing of different phases of human dispersal, occupation and technological innovation (and hence cognitive evolution) cannot be properly understood. Beyond the field of archaeology the work is also of relevance to Climate Science as some of the time intervals discussed here, for example the interglacial MIS 11c, are often cited as partial-analogues for the current interglacial. If, however, it is not possible to clearly identify deposits of such interglacials then their potential use as analogues is greatly diminished. The question that the work is seeking to address is, therefore, important, relevant and timely.

 The manuscript is very well-organised and presented. The authors have set out very clearly the rationale for the work that is being carried and introduce the study area well. The manuscript highlights very nicely the need for unique AAR stratigraphies to be produced at a regional scale as it is not possible (or reliable) to assume that the background factors that control rates of racemisation, primarily temperature history, are consistent between study regions. This is the underlying limitation which prevents the well-defined AAR stratigraphy of the British Isles from being simply imported into areas such as eastern Europe. That is to say that a temperate maritime climate such as the British Isles has higher mean annual temperatures at the present and, most likely, across the Quaternary than central and eastern Europe and this probably results in higher rates of racemisation in the former. The investigation of the role of different temperature regimes on AAR values is the underlying aim of this study and the paper effectively communicates that it is this variable which is likely to have the biggest impact on AAR values. As such the study sites presented here are divided into four regions, based on temperature regime, in order to investigate the impact of this factor. The data is very clearly presented, the figures are powerful and effective in the way that they convey the datasets and make it easy to understand the patterns that are presented.

 The interpretation of the data is sound and rigorous. The authors make a very good attempt at highlighting what the data can do and what its limitations are. In particular the authors are very clear about the limitations that the existing dataset has for resolving the “Holsteinian” issue. This issue is based around the uncertainty that exists of whether Holsteinian deposits should be correlated with MIS 11 or 9. In the British Isles a separation of MIS 11 and 9 deposits is possible on the basis of AAR analysis, although even in this record the difference in AAR values in opercula derived from these two warm phases is smaller than that found between any other consecutive temperate phases. The AAR data here leaves the question of MIS 11c and MIS 9 unresolved and the authors are very clear about the limitations of the data to resolve this issue.

 Overall, therefore, this is a very important and timely paper that is both robust, well-organised and well-argued. The only area of the study that I feel would benefit from some greater clarification/discussion is: 1) the rationale for placing sites into specific “regions” and 2) the discussion of the potential role of temperature in causing differences in the rate of racemisation. I think that these changes are very minor and require only some clarifications within the existing text and possibly to one figure. I would like the authors to consider these issues. I do not believe that they are fundamental to the overall interpretations presented here but believe that they may help with clarity in certain aspects of the paper.

 Regarding point 1 - The study identifies four different regions within which the sites are placed. It is argued that these different regions are separated on the basis of temperature regime and the logic for this is shown in the mean annual, summer and winter temperatures shown in Table 1. However, these regions span significant geographical areas and the sites within them are not clustered or evenly distributed, some of the sites in the “Central Poland” group are spatially closer to sites within the “East Poland and Lithuania” group than they are to others within the “Central Poland” group. As temperature regime is a continuum it is not clear to me why, from a temperature, perspective those sites in “Central Poland” that are closer to sites in “East Poland and Lithuania” should respond more to the temperature regime of the former rather than the latter. For example, does the site of Skolosvow in southeastern Poland really have more in common climatically with Valakampiai in Lithuania, nearly 800 km to the north, than it does with the site of Piotrlowice, approximately 200km to the west?  Also it is a little unclear what the temperature values quoted in Table 1 relate to. I believe that they come from modelled climate data that is extrapolated from observational data, but do they represent the temperature regime of a point in the centre of the modelled regions (in which case would it not be better to quote the range of mean, winter and summer temperature that is found across these regions to show the temperature range that these sites may experience?) or an average temperature across the modelled regions (in which case would it not be helpful to quote a standard deviation for mean, winter and summer temperature in these regions?). I feel it is unlikely that this would change the conclusions of the work but it would provide a more realistic representation of the range of the temperature regime found in each of the regions. I think this is exemplified by Figure 3 which shows single point data for mean monthly temperature in each of the study regions but, as outlined above, there must be temperature variation within each region and this should be presented in the form of uncertainties which reflect this variation. These uncertainties might be small but it would allow the readers to assess how "unique" or "variable" the climates of each region are. In summary I think it would be helpful to clarify the temperature variability of each region more precisely and this could be done by: 1) succinctly outlining more clearly the rationale for ascribing sites to a specific region (particularly where they could lie in closer proximity to neighbouring regions), 2) explaining whether the temperature data shown in Table 1 and Figure 3 represent data from point samples in the centre of each region or averages across the designated region (this could be added to the methodology), 3) to try to represent the temperature range found within each region graphically in Figure 3 by using error bars and 4) to state how significant the temperature variance within a region is compared to that found between regions. With regard to the last point I don’t know whether this will be significant or not but without quantifying the intra-region variance it is difficult to know whether the temperature regime of each region is substantially different (i.e. do the average temperatures of the warmest months of different regions lie outside of the uncertainties of other regions).

 Regarding point 2 – this follows on from the final comment regarding point 1, would it be possible to be a little clearer on the component of temperature that the authors believe might be controlling rates of racemisation? The authors highlight that it is likely that the increasingly continental climate that occurs as one moves further eastward is likely to produce lower rates of racemisation and, therefore, lower ratios for interglacials of comparable age. However, at present there is little discussion of what specific component of the temperature regime might do this. For example, the current mean annual temperature difference between Britain and Eastern Poland is effectively 3 degrees (Table 1). Is the temperature control on racemisation quantified well enough to calculate, if this difference is assumed to persist through time, what the difference in estimated AAR ratio values might be for MIS 5e (125,000 years ago) or MIS 11 (405,000 years ago)? It would be really useful for readers to know what difference a thermal regime of say 1 degree might make because, if this is minimal, then it is more likely that factors such as the extremity of winter temperatures (e.g. the number of days below freezing?) are more significant. This level of analysis may not be possible but I think it would be useful for readers to know whether the differences seen in AAR ratio values can be explained by a difference in 1 or 2 degrees of mean annual temperature difference or whether they require the major differences in winter temperature that are seen to occur.

 Despite these points (which are minor points of clarification) the manuscript is well-written, well-produced and contains a rich range of data that is clearly presented and rigorously interpreted. I think that it will make an important contribution to the subject area.

Is the study design appropriate and does the work have academic merit?

Yes

Is the work clearly and accurately presented and does it cite the current literature?

Yes

If applicable, is the statistical analysis and its interpretation appropriate?

Yes

Are all the source data underlying the results available to ensure full reproducibility?

Yes

Are the conclusions drawn adequately supported by the results?

Yes

Are sufficient details of methods and analysis provided to allow replication by others?

Yes

Reviewer Expertise:

Quaternary Climates, Landscapes and Stratigraphy particularly of European interglacials

I confirm that I have read this submission and believe that I have an appropriate level of expertise to confirm that it is of an acceptable scientific standard.

# Response

**Published:** 2026-05-01

**Authors:** Ellie Nelson

**Affiliations:** Department of Chemistry, University of York Department of Chemistry, York, England, UK

We would like to thank the reviewer for taking the time to review this manuscript, their kind words regarding the work, and their useful comments to improve the manuscript.

 We have therefore revised the manuscript and supplementary information following their suggestions. We agree with the reviewer that the clarification of the regions could be clearer, and that a expansion of the discussion of temperature and what might be controlling the effects of racemisation will improve the manuscript.

 The original groupings were based on the sites’ geographical localities of the Thuringian Basin, NE German Plain, Central Polish Lowlands, and Western Polasie. Sites that were analysed from this part of Europe but not in these localities were included in the aminostratigraphy which they were geographically closest to, with the caveat that this was provisional. However, we hadn’t coherently explained this approach in the text, and so we have taken on board both reviewer’s comments about the limited rationale for dividing this area of Europe into regional stratigraphies. This has been very helpful in enabling us to reconsider the approach to grouping into sub regions and we have explained and justified the new approach more clearly in the revised text. The new approach involves using temperature variables from ERA5 (Hersbach et al., 2023) – a climate reanalysis dataset that assimilates instrumental records and climate simulations to produce a global climate dataset for the last century – to group sites together into clusters with the most similar temperature conditions over instrumental timescales. This was done using a hierarchical clustering approach. The assumption is that sites with similar historical temperatures over instrumental timescales are likely to have similar temperature histories over Quaternary time scales. This produced six clusters, which were largely similar to the original attributions with some exceptions. The sites within the North German Plain have been separated into two clusters, one in the northwest and one in the southeast of the plain. The sites of Neumark Nord 1 & 2 and Uichteritz were grouped with the southeast North German Plain rather than the Thuringian Basin. The Polish sites were grouped together into the same clusters, as previously presented, and the two sites from the coldest regions (Komorniki and Valakampiai) have been grouped together into a separate cluster.   This new approach has little impact on the conclusions of this paper, but the regional aminostratigraphies are now defined by climate parameters rather than geographical location. We believe it is important to have regional aminostratigraphic frameworks in order for these datasets to be used to help constrain the age of additional sites in the future, as these can be more readily used to test the age of new material, but it may become apparent that some of these regional datasets could be also combined. Table 1 has been amended according to the reviewer’s suggestion and now includes the mean annual, winter and summer temperatures for each cluster of sites, along with the standard deviation. We agree that this makes the range of temperatures for each cluster clearer. We have removed Figure 3, which has now been replaced with the new Figure 5, which shows the correlation between extent of IcPD and climate variables. This has resulted in changes to the numbering of all figures in the paper. Many studies over the last few decades have determined that racemisation has an exponential relationship with temperature, so a 1°C difference where the burial temperatures are around 4°C will have much less of an impact on the rate than a 1°C difference at 25°C. This point we have now included within section 2.1, so that this is made clearer to the reader. We also agree with the reviewer that duration of the colder conditions is a critical factor in protein breakdown.  However, we are cautious in our interpretations, because as the reviewer postulates, the temperature control on racemisation at the temperatures that occur during burial is not understood well enough to effectively model the relationship. This is also complicated because the estimation of burial temperature is more challenging the further back in time we try to simulate. We believe that the impact of mean annual temperature and the persistence of colder conditions is now better demonstrated by the new Fig. 5 included in the paper.  This study is the first step to explore these relationships over a region with these differences in climate regimes, and in the future, better constrained ages and temperatures will significantly advance this.

# Reviewer report: Reviewer response for version 1

**DOI:** 10.21956/openreseurope.23597.r66950

**Published:** 2026-01-07

**Authors:** Darrell Kaufman

**Affiliations:** 1 Northern Arizona University, Flagstaff, Arizona, USA

Nelson and co-authors apply amino acid geochronology using snail opercula to develop aminostratigraphies for the eastern North European Plain, covering deposits from the last 1 million years. These chronological frameworks are used to confirm and reassess the ages of Middle and Late Pleistocene interglacial deposits, evaluate temperature-related differences in the extent of amino acid racemization, and assess the resolving capacity of the method for sequences deposited during a single interglacial periods.

 The study design is appropriate, and the conclusions are well supported by the data. The study presents an extensive and exceptionally high-quality amino acid dataset derived from resilient opercula biocarbonate that was subjected to rigorous bleaching pretreatment. The analyzed samples are linked to existing chronostratigraphic frameworks, with careful use of independent age controls and correlations. The extensive supplementary documentation details age assignments and regional pollen chronostratigraphies. The recovery of amino acids from specimens as old as Miocene highlights the integrity of the opercula as an archive for ancient biomolecules.

 One aspect that could be reconsidered is the decision to subdivide the dataset into four regional aminostratigraphies. Although temperature is clearly a major control on D/L values, it varies relatively continuously across space and can be addressed analytically without imposing discrete regional boundaries, which can be somewhat arbitrary. Multiple examples, including the North American Pacific Coast transect mentioned twice in the manuscript, demonstrate the value of examining D/L behavior along explicit climate gradients rather than partitioning sites into separate regions. If there is a clear analytical or interpretive advantage to grouping sites into regional aminostratigraphies rather than treating them along a continuum, articulating that rationale more explicitly would strengthen the presentation.

 This issue is particularly relevant given the stated aims of the paper. The Abstract states that the regional aminostratigraphies were used to explore D/L differences driven by contrasting temperature histories, and the text devotes substantial discussion to modern climate patterns and to D/L differences among coeval deposits across the study area. However, the manuscript currently lacks a figure that directly illustrates how D/L varies with temperature or continentality. While the extensive UK dataset provides a valuable reference framework, the use of horizontal iso–D/L lines in Figure 5 makes it difficult to identify the effect of temperature gradients. Sloping iso-chron lines, or regressions of D/L against temperature metrics (e.g., mean, maximum, or seasonal range), could offer a more direct test of the geographic trends in D/L and strengthen the paleotemperature interpretation.

 The framing of the goals of the amino acid geochronology in the Introduction could be better aligned with the Conclusions. The Introduction emphasizes confirmation of existing age correlations based on independent chronologic control, whereas the Conclusions highlight the use of amino acid geochronology to provide age constraints at sites with poor or uncertain age control. Clarifying early on that the data are used both to test existing correlations and to assign new or revised correlated ages where independent control is limited would better reflect the full scope of the study.

 The manuscript appropriately focuses on a single amino acid, alanine, which clearly captures the dominant age-related signal in the dataset, and the inclusion of results from other amino acids in the Supplementary material is welcome. However, the interpretive role of these additional amino acids is not fully articulated. It would be helpful to state explicitly whether they are used primarily as a consistency check on alanine-based interpretations, and to describe how discrepancies, if any, were evaluated or resolved. This clarification would help readers understand how the full amino acid suite informs the robustness of the conclusions.

 The Methods section notes the analysis of standards and procedural blanks, but these data are not presented. Including results for standards, blanks, plus the amino acid concentration measurements in the Supplementary material would be useful. In particular, given that several specimens yielded non-detectable FAA, reporting blank concentrations would help document the detection limits and may be valuable for future methodological comparisons.

 In sum, the manuscript presents a well-designed and carefully executed application of amino acid geochronology to develop aminostratigraphic frameworks for the eastern North European Plain. The study is supported by an exceptionally high-quality dataset, rigorous analytical protocols, and thoughtful integration with independent chronostratigraphic and palynological evidence. The comments and suggestions raised above are largely clarificatory refinements rather than fundamental concerns. Overall, this is a strong contribution to European Quaternary chronostratigraphy, and the paper should be accepted for formal indexing once the authors have reflected on these suggestions.

Is the study design appropriate and does the work have academic merit?

Yes

Is the work clearly and accurately presented and does it cite the current literature?

Yes

If applicable, is the statistical analysis and its interpretation appropriate?

Yes

Are all the source data underlying the results available to ensure full reproducibility?

Yes

Are the conclusions drawn adequately supported by the results?

Yes

Are sufficient details of methods and analysis provided to allow replication by others?

Yes

Reviewer Expertise:

My research area is relatively broad in the field of Quaternary geology, paleoclimatology and geochronology. I am an expert in amino acid geochronology, the topic of this paper.

I confirm that I have read this submission and believe that I have an appropriate level of expertise to confirm that it is of an acceptable scientific standard.

# Response

**Published:** 2026-05-01

**Authors:** Ellie Nelson

**Affiliations:** Department of Chemistry, University of York Department of Chemistry, York, England, UK

We would like to thank the reviewer for taking the time to review this manuscript, their kind words regarding the work, and their useful comments to improve the manuscript. We have therefore revised the manuscript and supplementary information following their suggestions.

 The reviewer is right that burial temperature is a critical consideration in aminostratigraphy, and so in this paper we had erred on the side of caution in our original groupings, feeling that it was better to initially compare sites within regions that demonstrably experience very similar present-day temperatures. The original groupings were based on the sites’ geographical localities of the Thuringian Basin, NE German Plain, Central Polish Lowlands, and Western Polasie. Sites that were analysed from this part of Europe but not in these localities were included in the aminostratigraphy which they were geographically closest to, with the caveat that this was provisional. However, we hadn’t coherently explained this approach in the text, and so we have taken on board both reviewer’s comments about the limited rationale for dividing this area of Europe into regional stratigraphies. This has been very helpful in enabling us to reconsider the approach to grouping into sub regions and we have explained and justified the new approach more clearly in the revised text. The new approach involves using temperature variables from ERA5 (Hersbach et al., 2023) – a climate reanalysis dataset that assimilates instrumental records and climate simulations to produce a global climate dataset for the last century – to group sites together into clusters with the most similar temperature conditions over instrumental timescales. This was done using a hierarchical clustering approach. The assumption is that sites with similar historical temperatures over instrumental timescales are likely to have similar temperature histories over Quaternary time scales. This produced six clusters, which were largely similar to the original attributions with some exceptions. The sites within the North German Plain have been separated into two clusters, one in the northwest and one in the southeast of the plain. The sites of Neumark Nord 1 & 2 and Uichteritz were grouped with the southeast North German Plain rather than the Thuringian Basin. The Polish sites were grouped together into the same clusters, as previously presented, and the two sites from the coldest regions (Komorniki and Valakampiai) have been grouped together into a separate cluster.   This new approach has little impact on the conclusions of this paper, but the regional aminostratigraphies are now defined by climate parameters rather than geographical location. We believe it is important to have regional aminostratigraphic frameworks in order for these datasets to be used to help constrain the age of additional sites in the future, as these can be more readily used to test the age of new material, but it may become apparent that some of these regional datasets could be also combined.

 The reviewer’s suggestion to use regressions of D/Ls against temperature metrics is an excellent idea, and a new figure (fig. 5) demonstrating how extent of IcPD compares to longitude, mean annual, mean winter and number of days under 2°C has been included. These were the temperature variables with the strongest correlation to IcPD: both variation in mean temperature, but also the number of days spent below temperatures where the rate of protein decomposition reduces significantly (or pauses) and hence influence the extent of IcPD observed. This supports our original conclusion that both magnitude and duration are important for defining aminostratigraphic regions. To emphasise the use of amino acid geochronology to provide age constraints at site with poor or uncertain age control we have added some text to the last paragraph of the introduction and first paragraph of the Material section (3.1) to explain that these new aminostratigraphies are also used to constrain the age of sedimentary deposits with uncertain independent age control. We actually use all of the amino acids for our interpretation, but agree that we need to clarify how we do this in the text. We have therefore expanded the explanation on how each amino acid is used to interpret the age signal within the data, and whether a closed system has been retained in each operculum. The explanation on the exclusion criteria has also been expanded to make it clearer how anomalous data points are identified and rejected. We have also made the rejection criteria clearer in the SI.  The SI data has been expanded so that all analytical replicates are presented, and all relevant amino acids present in each sample is presented with both the concentration of amino acid produced from reach replicate and the D/L value. We present now an average concentration and D/L value for the standards and blanks ran over the timeframe that this study was predominantly carried out (2022-2025).
